# Atrial fibrosis in atrial fibrillation: Mechanisms, mapping techniques and clinical applications

**DOI:** 10.1113/JP288680

**Published:** 2025-05-28

**Authors:** Caterina Vidal Horrach, Laura Bevis, Cynthia Nwanna, Alexander M Zolotarev, Mahmoud Ehnesh, Semhar Biniam Misghina, Sayed Al‐Aidarous, Shohreh Honarbakhsh, Caroline H. Roney

**Affiliations:** ^1^ School of Engineering and Materials Science Queen Mary University of London London UK; ^2^ Electrophysiology Department, Barts Heart Centre Barts Health NHS Trust London UK

**Keywords:** atrial fibrosis, atrial fibrillation, digital twins, electroanatomic mapping, fibrosis‐targeted ablation, magnetic resonance imaging

## Abstract

Atrial fibrosis plays a pivotal role in the initiation and progression of atrial fibrillation (AF), creating a substrate for AF through structural, electrical and functional remodelling. Atrial remodelling results from various factors, including inflammation, obesity, hypertension and ischaemia, which collectively disrupt cellular coupling and ion channel function. The heterogeneity formed by the distribution of atrial fibrosis creates a substrate for abnormal electrical propagation and arrhythmias through alterations in ionic currents and conduction slowing. The extent of atrial fibrosis may be investigated through multiple modalities, including imaging and electroanatomic mapping. The pathological processes underlying atrial fibrosis are exacerbated in the transition from paroxysmal to persistent AF, highlighting the need for advanced diagnostic and therapeutic strategies. In this review, we cover the role of atrial fibrosis in AF, evaluate the modalities used to quantify and characterize atrial fibrosis, giving an overview of their clinical applications in stratifying patients and guiding treatment strategies, and discuss the integration of fibrosis information in computational AF models. We explore how the combination of experimental and computational techniques can enhance our understanding of the arrhythmogenic effects of fibrosis and the challenges inherent in translating mechanistic insights into effective therapies.

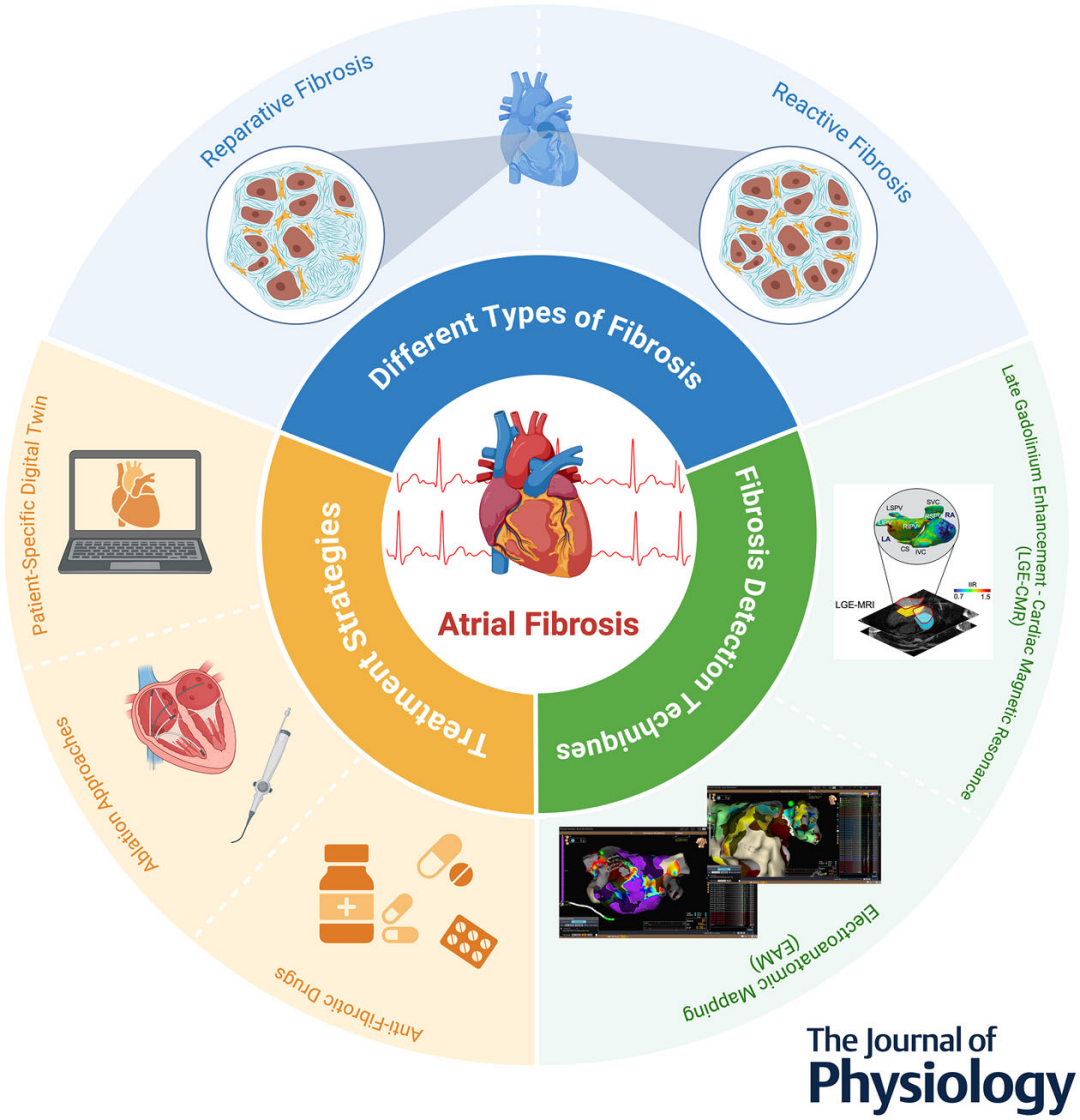

## Graphical abstract

**Abstract figure legend** Summary of key aspects of atrial fibrosis in atrial fibrillation, highlighting atrial fibrosis as a hallmark of atrial fibrillation (AF). (1) Different types of fibrosis (blue), illustrating reparative and reactive fibrosis; (2) fibrosis detection techniques (green), illustrating late gadolinium enhancement cardiac magnetic resonance (LGE‐CMR) and electroanatomic mapping (EAM) as key methods for fibrosis assessment; and (3) treatment strategies (orange), summarizing anti‐fibrotic drugs, ablation approaches and patient‐specific digital twin models. This review explores the mechanisms, mapping techniques and clinical applications of atrial fibrosis in AF.

## Role of fibrosis in atrial fibrillation (AF)

### Pathophysiology of atrial fibrosis

**Figure 1:**
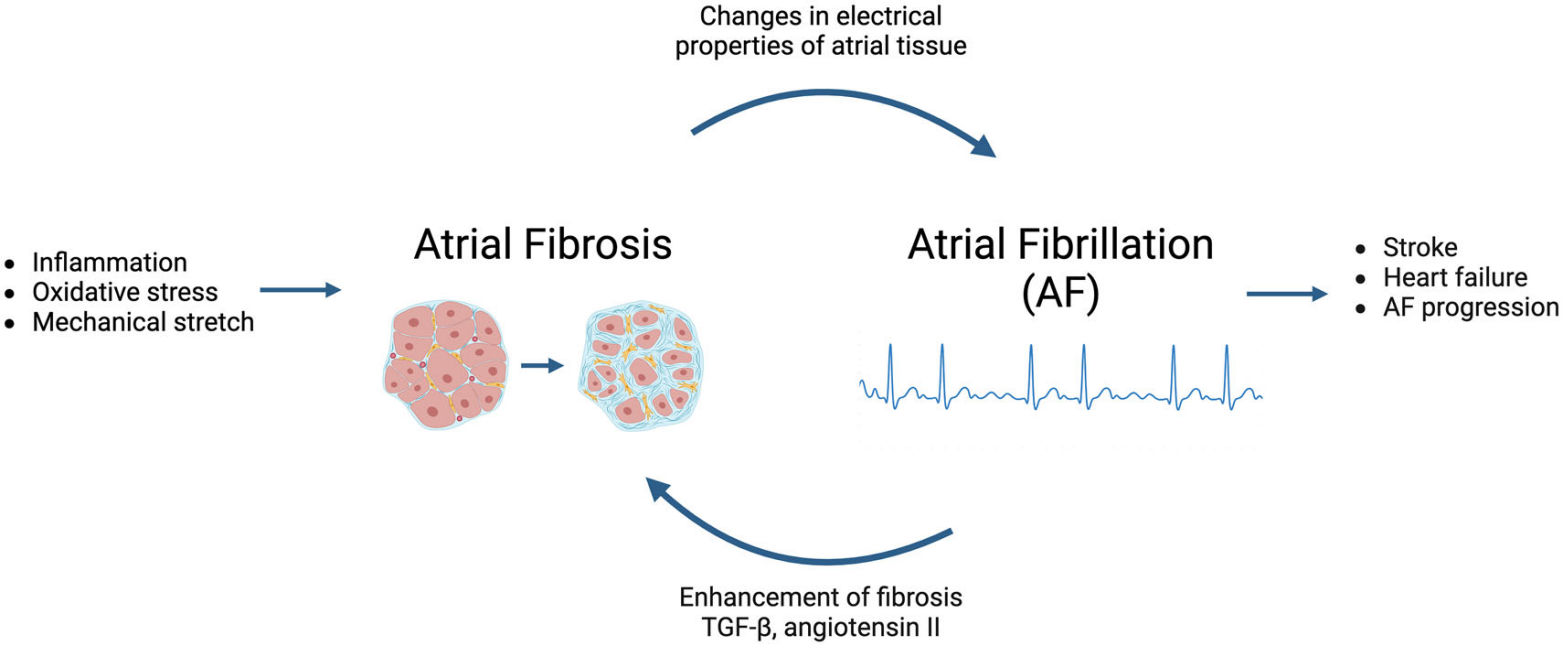
Pathophysiology of atrial fibrosis in AF An overview of the different factors contributing to atrial fibrosis in patients with AF, which can result in stroke, heart failure or AF progression. Key drivers of atrial fibrosis are inflammation, oxidative stress and mechanical stretch, which creates a cycle as fibrosis is enhanced by AF through different signalling pathways.

Atrial fibrosis refers to the excessive build‐up of extracellular matrix (ECM) components resulting in structural remodelling of the atria. This causes a disruption in the electrical conduction pathways, which promotes the initiation and maintenance of AF (Dzeshka et al., 2015; Harada & Nattel, 2021; Leventopoulos et al., 2023). This process is frequently initiated by inflammatory mediators, oxidative stress and mechanical stretch, producing a perpetuating cycle where AF additionally enhances fibrosis through several different signalling pathways, such as transforming growth factor beta (TGF‐β) and angiotensin II (Nattel, 2017) (Fig. 1). The pathophysiology of atrial fibrosis involves several cellular and signalling mechanisms.

Cardiac fibroblasts are the primary cells responsible for fibrosis in AF, becoming activated by several stimuli, such as inflammatory cytokines, growth factors such as TGF‐β and platelet‐derived growth factor, and mechanical stretch. The activation of these cells leads to enhanced collagen production and excessive deposition of the ECM, contributing to the fibrotic process. Additionally, activated fibroblasts can differentiate into myofibroblasts, which have contractile characteristics and play a significant role in tissue remodelling (Frangogiannis, 2021; Schotten et al., 2025; Sohns & Marrouche, 2020).

The TGF‐β signalling pathway is considered the principal driver of fibrosis in AF. It enhances fibroblast proliferation and collagen synthesis, significantly contributing to atrial remodelling. The renin–angiotensin–aldosterone system also plays an important role, with angiotensin II stimulating fibroblast activation and fibrosis through specific receptors. Furthermore, oxidative stress from reactive oxygen species activates additional signalling pathways that result in elevated collagen production and fibroblast activity. Together, these pathways reinforce the fibrotic activity, enhancing AF progression (Jalife & Kaur, 2015; Li et al., 2020; Nattel, 2017).

### Different types of fibrosis

**Figure 2:**
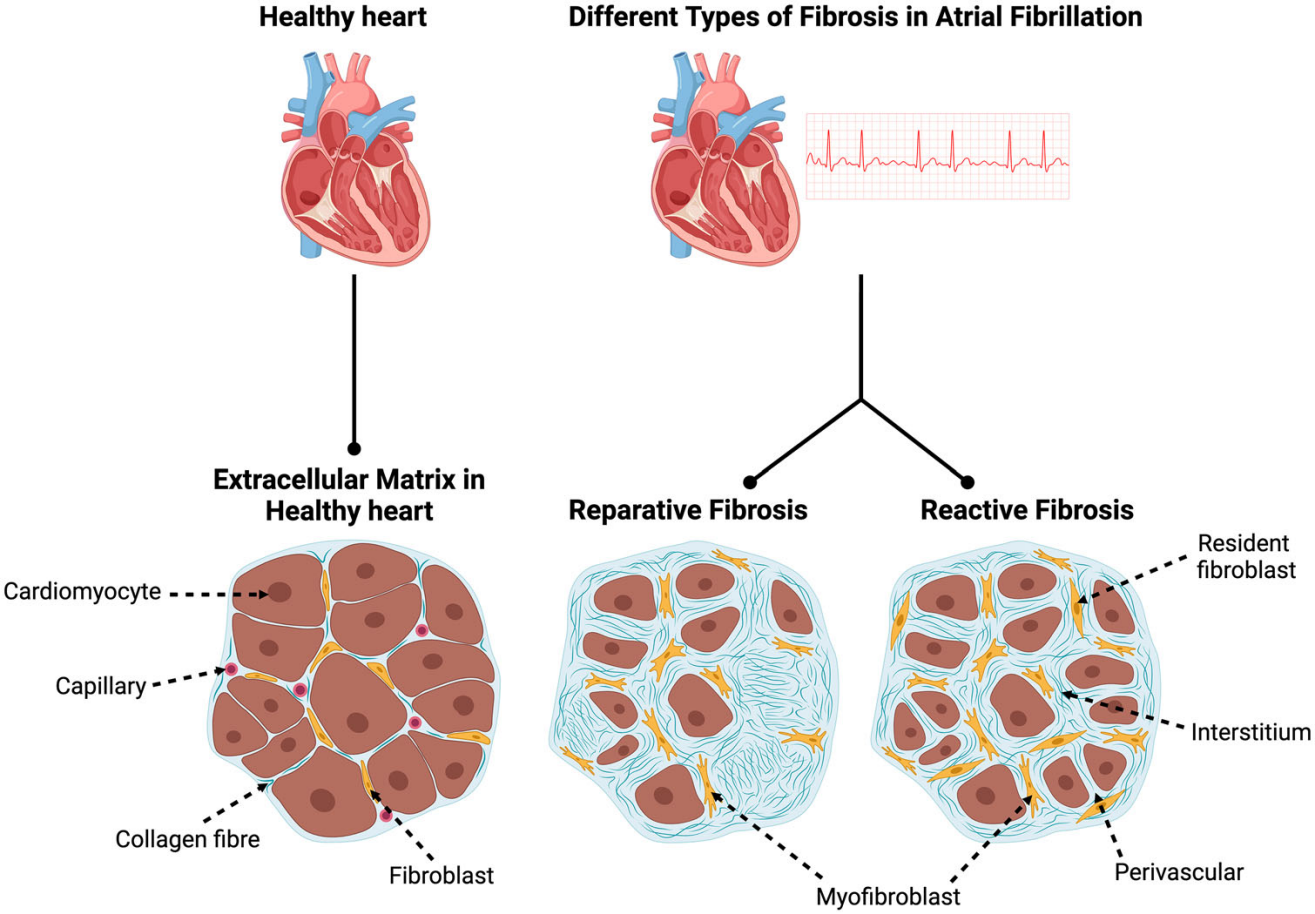
Different types of cardiac fibrosis: reparative fibrosis and reactive fibrosis Left: 3‐D extracellular matrix network of collagen fibres that support cardiac cells (cardiomyocytes, capillaries and fibroblasts). Reparative fibrosis (middle) appears as a 3‐Dextracellular matrix with collagen‐based scar that is produced through a healing process, replacing dying cardiomyocytes. Reactive fibrosis (right) is associated with heart failure caused by pressure overload and is characterized by widespread deposition of cross‐linked collagen in interstitial and perivascular regions.

There are two main types of atrial fibrosis: reactive fibrosis and reparative fibrosis. Reactive fibrosis occurs as a result of pressure overload or cardiac inflammation, whereas reparative fibrosis occurs after apoptosis and is identified by the replacement of dead tissue with collagenous scar tissue (Burstein & Nattel, 2008; Verheule & Schotten, 2021) (Fig. 2). Both types of fibrosis interfere with the electrical conduction within the atria resulting in the development and progression of AF (Li et al., 2020). It is crucial to recognise that these two different types of fibrosis are not mutually exclusive, and patients can have a combination of both types of fibrosis in the atria (Nattel, 2017).

Within reactive fibrosis, fibroblasts proliferate and change into myofibroblasts, resulting in a build‐up of ECM material. Primarily, in response to pressure overload, reactive fibrosis exhibits as perivascular fibrosis and gradually advances to interstitial fibrosis. Reactive fibrosis includes perimysial fibrosis, which occurs around the muscle bundles, and endomysial fibrosis, which is a thickening of the normal endomysial collagen septae between cardiomyocytes (Fig. 2). During perimysial fibrosis, the increase in collagen causes the thin fibrous layers surrounding the muscle bundles to thicken, whereas the muscle bundles themselves preserve their structure. In some cases, thick interstitial collagen strands can accelerate conduction rather than slow atrial electrical conduction (de Sensi et al., 2021). This fibrotic remodelling process involves ECM synthesis and the combination of collagen and dysregulation of ECM turnover through matrix metalloproteinases and their inhibitors. These structural and biochemical alterations can lead to the persistence and progression of AF (de Boer et al., 2019; Nattel, 2017).

Endomysial fibrosis refers to a particular form of scarring in the atrial tissue, where collagen accumulates between myocytes, interfering with electrical conduction pathways, and contributing significantly to the abnormal heart rhythms observed in AF (Maesen et al., 2022). As endomysial fibrosis progresses, it causes a loss of normal muscle fibres and increases atrial stiffness, making the atria more prone to arrhythmias (Karakasis et al., 2024). The fibrotic tissue also produces permanent anatomical substrates for AF, contributing to its persistence and making it harder to revert to normal sinus rhythm (SR) (Maesen et al., 2022; Winters et al., 2022). Unlike overall atrial fibrosis, it is the primary factor of conduction disturbances, disrupting transverse muscle connections and promoting irregular electrical wave propagation causing re‐entrant circuits that sustain AF. Understanding different types of atrial fibrosis, including endomysial fibrosis, can lead to more effective therapies for managing the progression of fibrosis and prevention in AF.

By contrast, reparative fibrosis occurs when fibrous tissue replaces dead cardiomyocytes, a process often seen in myocardial infarction scar. Reparative fibrosis is important because it stabilizes the necrotic tissue deficiency left by the infarction. When focusing on AF, extensive apoptosis results in areas of fibrosis that disrupt muscle bundles, producing clear longitudinal conduction barriers. Generally, reparative fibrosis is irreversible, and preventing reparative fibrosis may rely more on delaying cardiomyocyte death rather than inhibiting fibroblast proliferation (de Boer et al., 2019; Nattel, 2017).

Transmural fibrosis is a more advanced type of fibrosis, especially in the subepicardial and midwall layers. This type of fibrosis extends through the entire thickness of the atrial wall and can result in conduction disturbances and developing a higher risk of AF (Eckstein et al., 2013; Ravelli et al., 2023). This scarring across the atrial wall can result in significant conduction block, slowing electrical signals, which can cause the formation of re‐entry circuits sustaining AF. Additionally, transmural fibrosis causes electrical heterogeneity, which may cause AF to be more persistent.

Many conditions contribute to the development of atrial fibrosis. Hypertension influences chronic mechanical stress on the atria, stimulating fibrotic remodelling. Similarly, heart failure identified by left ventricular dysfunction can lead to atrial dilatation and promote fibrosis through elevated pressure. Another factor contributing to atrial fibrosis is inflammatory cells. These cells lead to cytokines being released, which activate fibroblasts and enhance ECM deposition. Age‐related changes play an important role because ageing alterations in the ECM can increase susceptibility to fibrotic remodelling (Harvey et al., 2016; Li et al., 2020).

### Arrhythmogenic effects of fibrotic remodelling

**Figure 3:**
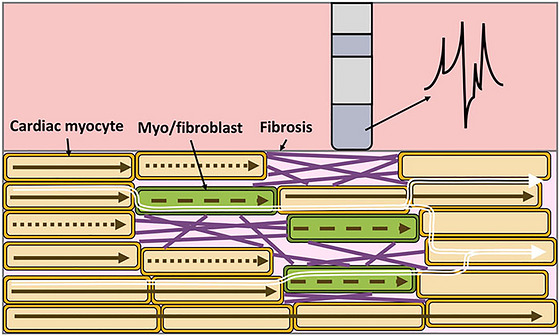
Electrical propagation in heterogeneous fibrotic cardiac tissue Electrical propagation in heterogeneous fibrotic cardiac tissue, comprising cardiomyocytes (orange), myofibroblasts/fibroblasts (green) and collagen fibres (purple). Excitation propagates left to right (brown arrows), with conduction block (dotted arrows) and slowed conduction (dashed arrows). The resulting zigzag wavefront (white arrows) prolongs the unipolar electrogram and introduces asymmetry with multiple deflections (Sánchez & Loewe, 2022)

Fibrotic remodelling in atrial tissue plays an important role in the development of arrhythmias, specifically in AF, by disrupting normal electrical conduction pathways. For example, the deposition of non‐conductive scar tissue results in irregular impulse propagation. Fibrosis disrupts normal atrial conduction properties by disrupting the normal myocardial architecture, leading to a slow, discontinuous signal transmission with ‘zigzag’ propagation (Fig. 3) as a result of reduced regional coupling (de Bakker et al., 1993; Gardner et al., 1985). This variation in conduction velocity (CV) fosters irregular wave patterns and re‐entry circuits, increasing the risk of AF (Burstein & Nattel, 2008). Animal studies indicate that increased interstitial fibrosis enhances conduction heterogeneity (Kawara et al., 2001; Li et al., 1999). A dog model revealed that atrial fibrosis causes localized conduction slowing and increases conduction heterogeneity, thus forming the basis for unidirectional conduction block and macro re‐entry (Burstein & Nattel, 2008), which are essential for AF initiation and perpetuation. Also, many studies have shown a relationship between reduced expression of Cx43 and enhanced fibrosis (Trovato‐Salinaro et al., 2006; van Veen et al., 2005), which suggests that fibrotic regions reduce connexin‐43 expression, impairing gap junctional communication and further promoting conduction anisotropy. These structural disruptions facilitate a proarrhythmic substrate that promotes AF progression from paroxysmal to persistent forms (Vlachos et al., 2016).

In addition, fibrotic remodelling triggers ectopic activity by disrupting the protective source–sink mismatch that normally prevents small groups of misbehaving myocytes from triggering propagating impulses (Sánchez, Trenor et al., 2021). In healthy myocardium, spontaneous calcium waves or delayed afterdepolarizations in isolated myocytes fail to generate detectable voltage changes unless a critical mass of myocytes depolarizes synchronously (Voigt et al., 2012).

AF maintenance is influenced by several mechanisms involving atrial fibrosis. It disrupts normal electrical conduction by forming fibrotic tissue that slows impulse propagation and re‐entry circuits (Nguyen et al., 2014; Verheule & Schotten, 2021). Fibrosis alters the expression of ion channels and affects the myocyte action potential, increasing the susceptibility to ectopic foci especially in the pulmonary veins, causing AF maintenance (Karakasis et al., 2024; Schotten et al., 2025). Inflammation also plays a role in promoting fibroblast activation collagen deposition (Nattel, 2017). Furthermore, atrial fibrosis can lead to atrial dilatation, increasing the complexity of re‐entry circuits because of the increase in the atrial surface area (de Groot et al., 2025). These different mechanisms that affect atrial fibrosis contribute to AF maintenance.

To gain a better understanding of the relationship between atrial fibrosis and AF, significant insight has been provided when using advanced techniques such as optical mapping, high‐resolution magnetic resonance imaging (MRI) and histology. One study highlighted the limitations of current AF treatments and the potential 3‐D computer models to optimize ablation strategies, demonstrating that human‐derived models could improve the personalization and effectiveness of AF treatment approaches (Zhao, Kharche et al., 2015). Follow‐up research further demonstrated how fibrosis affects electrophysiological properties, with high‐resolution MRI revealing detailed structural changes (Zhao, Hansen et al., 2015). Furthermore, Hansen et al. (2017) revealed that intramural re‐entry, anchored to fibrosis‐insulated atrial bundles, drives sustained AF. This research highlights how 3‐D structural functional mapping exhibits significant differences in endocardial and epicardial activation, indicating the important role of intramural re‐entry in AF. Additionally, further work highlights the role of fibrotic tissue in altering conduction pathways, providing important information into the mechanisms that drive fibrosis in AF in human hearts (Hansen et al., 2017; Zhao et al., 2017). Overall, this research emphasizes the significance of fibrotic infiltration in AF pathophysiology and the importance of advanced imaging techniques when studying these alterations.

Atrial fibrosis plays a significant role in the development of endo‐epicardial dissociation (EED) by disrupting the structural and electrical coupling between the endocardium and epicardium leading to an increase in fibrillation complexity. Fibrosis, particularly in the epicardial layer, disrupts electrical connection between muscle bundles, causing both intra‐epicardial and EED. It also impairs transmural conduction, further developing EED. Additionally, endomysial fibrosis in the epicardial layer leads to loss of continuity, reducing the synchronizing effect and increasing the complexity of fibrillatory conduction (Gharaviri et al., 2020; Verheule et al., 2014). A study by Eckstein et al. (2013) investigated the mechanisms underlying breakthrough events during AF, focusing on the role of transmural conduction and EED. The study demonstrated how fibrosis can disrupt electrical coupling between endocardial layers, enhancing EED, resulting in asynchronous activation and promoting AF persistence. The study also demonstrated how the presence of fibrosis in the epicardial layer is associated with higher incidence of transmural conduction breakthrough, further contributing to the complex activation patterns observed during AF (Eckstein et al., 2013).

Experimental models of EED have been fundamental in informing different theories of AF maintenance, especially in relation to breakthroughs driven by fibrosis infiltration. These animal models provide critical insights into the complex mechanisms underlying AF and have motivated the development of bilayer computational models that incorporate interactions between the endocardium and epicardium. A study by Parameswaran et al. (2020) demonstrated significant heterogeneity in dissociation during fibrillation in patients with persistent AF, highlighting the need for simultaneous endo‐epicardial mapping. Additionally, Verheule et al. (2014) reported that epicardial fibrosis disrupts electrical connections, promoting dissociation and increasing AF stability. Computational models have revealed that fibrosis density and weaker interlayer coupling promote breakthrough waves, enhancing AF maintenance (Zakeri Zafarghandi & Jacquemet, 2024). Similarly, Gharaviri et al. (2020) found that epicardial fibrosis increases dissociation and breakthrough waves. Finally, Chen et al. (2018) revealed that intramural fibrosis and dissociation sustain AF, highlighting the importance of structural remodelling.

Furthermore, fibrosis develops along the anisotropic fibre orientation of the atria, creating conduction heterogeneity and promoting re‐entry circuits. Alteration of fibre orientations result in decreased anisotropy, indicating a disruption in the organized alignment of fibres in the atria, and slower conduction speeds as a result of these fibrotic regions (Kwan et al., 2025). Additionally, in fibrotic tissue, increased fibre direction heterogeneity results in greater angle differences between neighbouring fibres (Ho, 2002; Kwan et al., 2025; Palacio et al., 2021). In fibrotic areas, modified conduction directions may enable the development of unidirectional conduction block (Kwan et al., 2025).

### Correlation between fibrosis and AF progression: paroxysmal *vs*. persistent AF

The impact of fibrosis is particularly prominent in patients with persistent AF, where it significantly alters the atrial substrate compared to those with paroxysmal AF (PAF). Studies have shown that persistent AF patients have lower left atrial voltage compared to PAF patients, even after adjusting for indexed left atrial volume (Fiala et al., 2009; Starek et al., 2023; Verma et al., 2015), suggesting increased atrial fibrosis. Moreover, patients with persistent AF exhibit a greater degree of atrial fibrosis, as demonstrated by late gadolinium enhancement on cardiac magnetic resonance imaging (LGE‐CMR) (Daccarett et al., 2011).

In persistent AF, fibrosis leads to heterogeneous conduction, which disrupts the uniform propagation of electrical impulses across the atria. This creates slow conduction corridors and areas of conduction block, contributing to increased wavefront curvature and the formation of pivot points, all of which facilitate the development and stabilization of re‐entrant circuits (Frontera et al., 2022). As AF persists, the fibrotic remodelling further amplifies conduction abnormalities, increasing the number of re‐entrant rotational activities and the expansion of areas maintaining these circuits. This results in a greater number of patients exhibiting re‐entry activity outside the pulmonary veins (Lim et al., 2017). These structural changes in the atrial substrate contribute to the reduced efficacy of pulmonary vein isolation (PVI) alone in persistent AF because the arrhythmia becomes less reliant on triggers from the pulmonary veins and more dependent on the fibrotic substrate for its maintenance.

## Mapping techniques for detecting fibrosis in AF

Cardiac fibrosis quantity and distribution can be assessed *ex vivo* using histological analysis, which is the gold standard method of assessment. However, this cannot be performed *in vivo*, and so cardiac mapping technologies are used to provide a surrogate method of assessment that can be used in the clinical environment. Specifically, cardiac mapping is an important technique used to produce detailed visualisations of the electrical activity or structural properties of the heart, which enable identification of features such as fibrosis in AF (Magtibay et al., 2019). There are several types of mapping techniques that can be performed invasively [e.g. electroanatomic mapping (EAM) during catheter ablation] or non‐invasively (e.g. imaging‐based mapping such as LGE‐CMR) (La Rosa et al., 2021). Recent developments in new high‐resolution imaging techniques, development of new catheters, computational tools and machine learning methods have significantly enhanced the precision and resolution of cardiac mapping in atrial fibrosis detection. The understanding of such mapping techniques is important as it can improve the prediction of treatment outcomes in patients with AF and guide therapy selection (Heijman et al., 2021). This section provides an overview of histological analysis of fibrosis, followed by the main cardiac mapping techniques used to detect fibrosis in AF. Finally, we discuss blood biomarkers of atrial fibrosis, which provides another method for assessing the presence of fibrosis in the clinical setting.

### Histological analysis of fibrosis

Histological analysis is performed *ex vivo* to quantify structural remodelling, by assessing factors such as fibrosis extent, intercellular space, myofibrillar loss, adipocyte extent, myocyte size and myocardial nuclear density (Takahashi et al., 2023; Yamaguchi, 2025). Histologically identified fibrosis is categorised into two main forms: interstitial fibrosis and replacement fibrosis, based on its distribution. Interstitial fibrosis involves the accumulation of ECM proteins in the myocardial interstitial space, typically with minimal cardiomyocyte loss (Disertori et al., 2017). Istrătoaie et al. (2015) observed fibroblast proliferation and collagen deposition in fibrotic regions, which is indicative of ongoing remodelling processes that contribute to the development of atrial fibrosis (Istrătoaie et al., 2015). Microvascular changes are also a key histological feature of interstitial fibrosis, with significant reductions in capillary density in these regions. This methodology provides a gold standard assessment that other techniques should be compared to; however, it can only be applied *ex vivo* on tissue samples, and so is not applicable in the clinical environment (Istrătoaie et al., 2015). Histological analysis of collagen type I staining in fibrotic hearts identified four fibrotic patterns: interstitial (collagen between cells), compact (dense collagen with no cardiomyocytes), diffuse (short fibrosis stretches) and patchy (long collagen fibres between myocardial bundles (de Jong et al., 2011).

### LGE‐CMR

LGE is an important tool in CMR for tissue identification and diagnosing cardiomyopathies. The extracellular gadolinum‐based contrast has slower wash‐in wash‐out time in tissues with increased extracellular space, enhancing these regions in CMR images taken later after its administration (Siebermair et al., 2017). Because fibrotic myocardial tissue has increased extracellular space compared to healthy myocardium, this leads to the enhancement of fibrotic regions in the atria, and their identification by higher signal intensities in LGE‐CMR images. Although LGE is not specific to fibrosis, LGE‐CMR intensity has been shown to correlate with the extent of fibrosis measured in surgical biopsy specimens (Karamitsos et al., 2020) and is generally considered a valid marker of myocardial fibrosis in cardiac conditions. Specific presentations of LGE‐CMR additionally allow for differential diagnosis between ischaemic and non‐ischaemic heart disease, which can aid diagnosis of different cardiomyopathies (Aquaro et al., 2023; Siebermair et al., 2017).

#### Quantifying thresholds of fibrosis and its challenges

**Figure 4:**
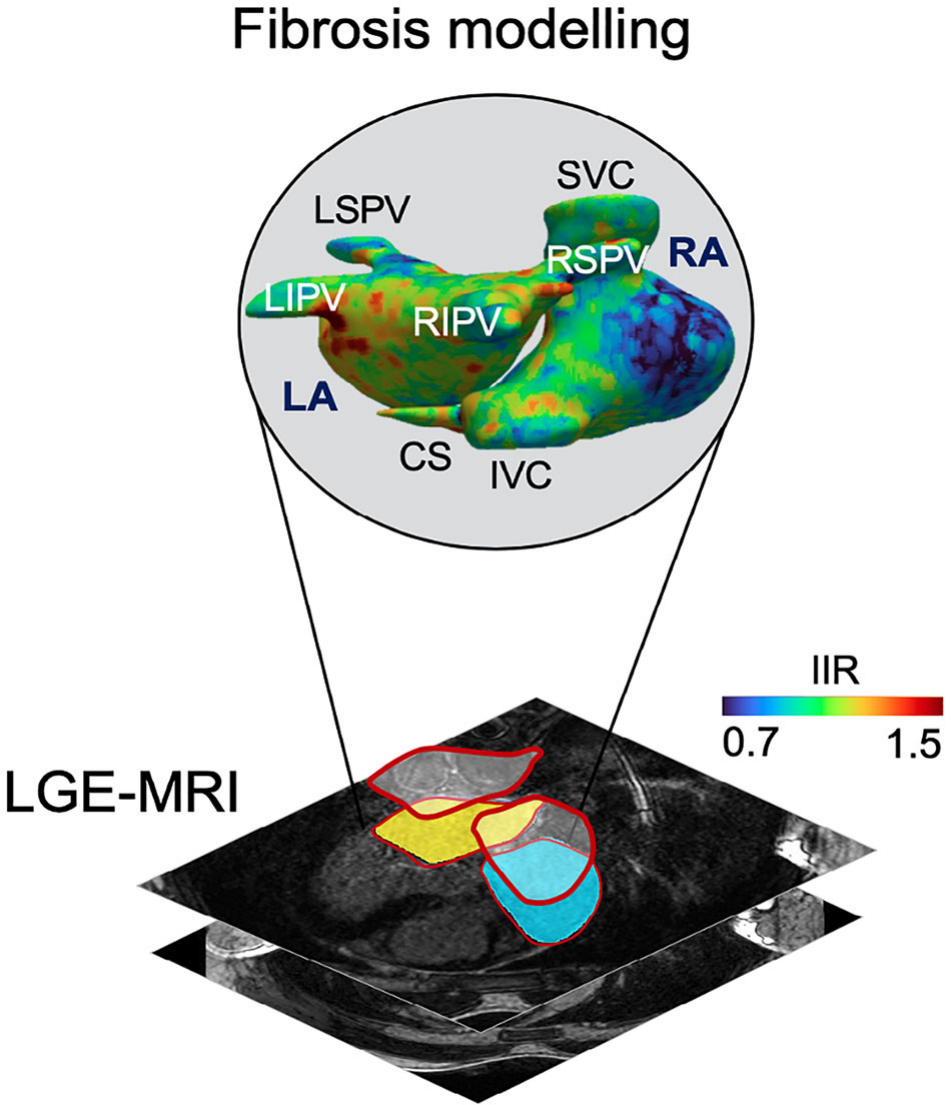
Registration of LGE‐MRI data for incorporating scar tissue into biatrial models Fibrotic masks were generated by mapping normalised blood pool intensities from LGE‐MRI onto the models, classifying regions based on image intensity ratio (IIR).

Identification of fibrosis from atrial LGE‐CMR images requires post‐processing to categorise tissue regions as fibrotic or non‐fibrotic, depending on the voxel intensity (Siebermair et al., 2017) (Fig. 4). The use of threshold‐based methods (Pontecorboli et al., 2016) and deep learning techniques (Razeghi et al., 2020) can improve reproducibility and accuracy; however, there is no clear consensus on appropriate thresholds for this categorisation (Jamart et al., 2020). The image intensity ratio (IIR) approach by Khurram et al. (2014) is the most common method (Khurram et al., 2014). Several studies have investigated thresholds to align fibrosis quantification by the IIR method with EAM data; for example, Khurram et al. (2014) identify IIR values of IIR > 1.61 as corresponding to fibrotic tissue defined by bipolar voltage mapping (voltage < 0.1 mV) and Zghaib et al. (2018) propose IIR > 1.22 as an effective threshold for scar detection. Other studies based on LGE‐CMR in healthy volunteers have proposed IIR thresholds of around IIR > 1.2 to define fibrotic tissue (Bertelsen et al., 2020). Recent studies have introduced optimised LGE‐MRI post‐processing methods to refine intensity thresholds, particularly for the anterior atrial wall, enhancing concordance with EAM (Nairn et al., 2023), and investigated different thresholds based on standard deviation (SD) values from the blood pool mean intensity to delineate fibrotic regions and dense scar (Andreasen et al., 2020; Malcolme‐Lawes et al., 2013; Pontecorboli et al., 2016). LGE‐CMR has significant clinical application, including patient selection and stratification for AF treatment and guiding ablation procedures.

Histological validation studies have demonstrated that thresholds set at 3–3.3 SD above the mean blood pool intensity provide reliable identification of fibrotic tissue (Pontecorboli et al., 2016). The variability in threshold selection across studies highlights the challenges in standardising IIR and SD‐based thresholds for fibrosis quantification.

In addition to debated thresholding methods, accurate quantification of atrial fibrosis using LGE‐CMR remains a significant challenge because of variability in imaging protocols and patient‐specific factors. Differences in scanner magnetic field strength, contrast agents and the timing of image acquisition can significantly influence intensity ratios, affecting fibrosis assessment. Additionally, technical parameters such as inversion time, patient heart rate and rhythm, contrast clearance rates, body mass index and blood oxygenation contribute to inconsistencies in threshold selection (Siebermair et al., 2017). Beyond these technical challenges, fibrosis detection in AF patients presents additional difficulties due to the thin atrial walls and the limited spatial resolution of LGE‐CMR, which hinder precise delineation of fibrotic tissue. Manual segmentation, which remains a widely used approach, introduces a degree of subjectivity, further complicating reproducibility (Longobardo et al., 2014; Siebermair et al., 2017). Automated segmentation tools and further advancements in imaging technology and clinical trials will remove this variability and help to optimise the role of LGE‐CMR in AF management.

To mitigate these challenges, Nairn et al. (2023) proposed the Estimated Optimized Image Intensity Threshold (EOITT) method. This approach improved concordance between LGE‐CMR and low‐voltage substrate diagnosis from electroanatomical mapping, and enhanced the prediction of rhythm outcomes after PVI. Although the EOIIT method demonstrated improved sensitivity and specificity, there remained inconsistencies with EAM, especially on the posterior wall, emphasizing the need for further standardization to improve reproducibility and clinical application. Sim et al. (2019) demonstrated reproducibility of atrial fibrosis assessment using an open‐source platform. Novel developments in imaging modalities are discussed in the ‘Future directions’ section of the review.

### EAM system

**Figure 5:**
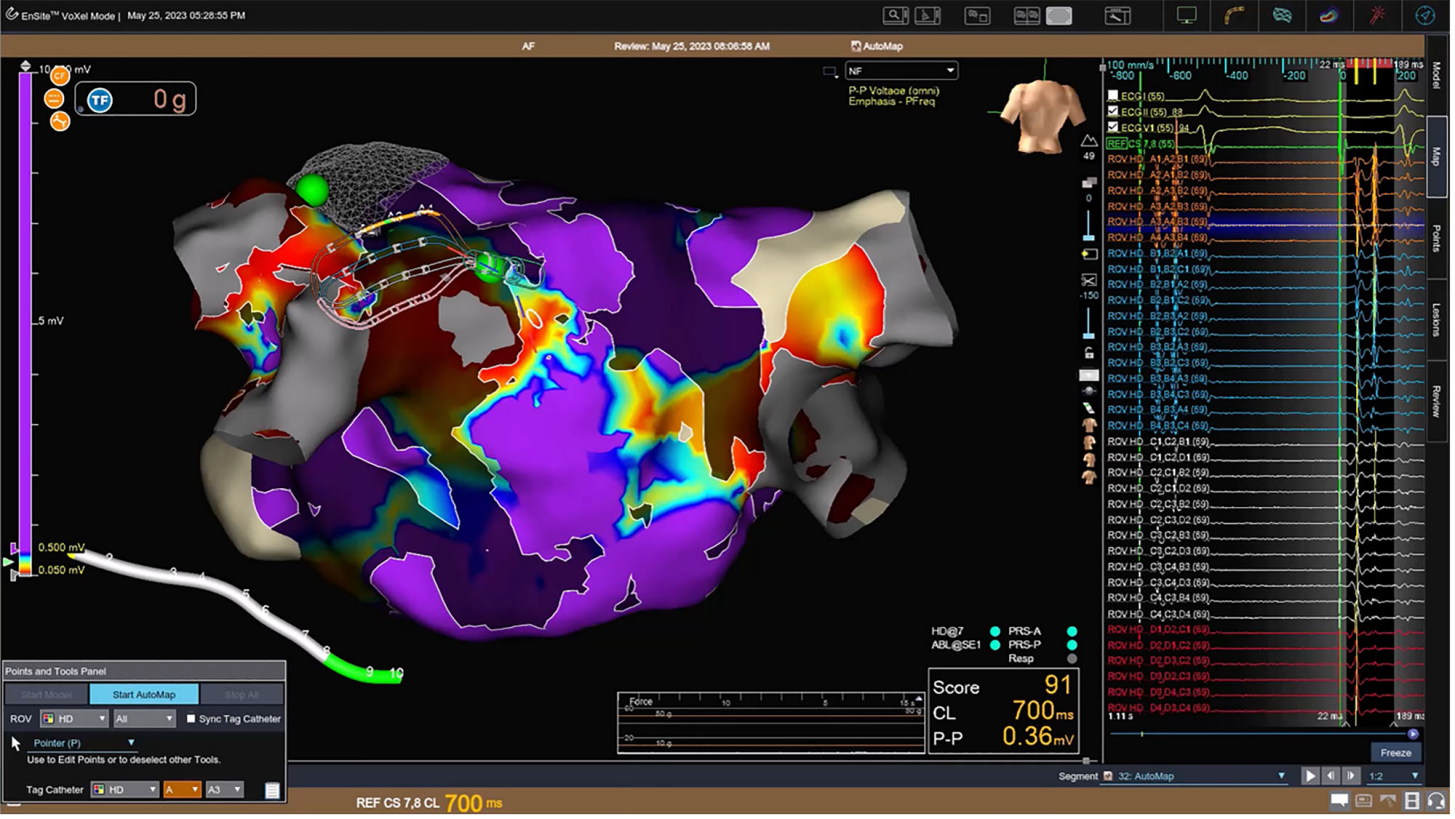
Representation electroanatomic mapping system (EAM) on left atrium geometry The system presented is EnsiteX (Abbott). Demonstrates layering of frequency map onto voltage map. Voltage map with emphasis of areas with signals greater than 250 Hz. Areas below 250 Hz are de‐emphasized or indicated by a faded colour.

Electroanatomic mapping provides detailed anatomical, structural and functional cardiac information using electrical recordings from intracardiac catheters (Fig. 5). Atrial fibrosis may cause regions of lower electrogram voltage, slower conduction or conduction heterogeneities, which can be identified using EAM. Specifically, EAM recordings capture activation rates (Jarman et al., 2012), conduction patterns (Cantwell et al., 2015), speed (Weber et al., 2010), wall thickness (Abeln et al., 2021) and voltage (Haldar et al., 2017), which are processed to assess scalar fields such as dominant frequency, activation time, peak‐to‐peak voltage and electrogram fractionation (Nademanee et al., 2004). Spatial voltage maps are derived from peak‐to‐peak amplitudes by interpolating the EAM data over the atrial anatomy, and can identify fibrotic regions, with bipolar voltage mapping being a gold standard for assessing fibrosis and scar tissue (Butcher et al., 2023). In addition to considering the amplitude of an electrogram, the morphology of electrograms may elucidate important information on electrical wavefront propagation and on features of the underlying tissue including the presence of atrial fibrosis. However, it is challenging to identify the mechanisms underlying electrogram morphology (Narayan et al., 2011).

#### Voltage mapping

##### Unipolar mapping

Although bipolar voltage is commonly used as a marker for atrial fibrosis, several studies have explored the use of unipolar voltage amplitude for substrate assessment. For example, Ye et al. (2024) used unipolar electrogram mapping during SR to identify conduction slowing, finding that areas with fractionated and low‐voltage unipolar signals exhibit slower local CV potentially indicating regions of fibrotic tissue. However, fractionation may result from other factors, limiting the clinical utility of these metrics. Additionally, Nairn et al. (2020) showed a strong correlation between bipolar and unipolar voltage maps in both SR and AF, providing unipolar thresholds equivalent to commonly used bipolar thresholds for identifying regions of fibrotic tissue.

##### Bipolar mapping

Low bipolar voltage measured on the atrial endocardium is commonly used as an indicator atrial fibrosis. The spatial distribution of these voltage values varies depending on disease severity. For example, Marcus et al. (2007) found that AF patients have more low‐voltage areas (LVAs) on the septal and posterior walls. Wavefront direction dependence seen in bipolar mapping further influences these voltage measurements, adding complexity to their interpretation (Haldar et al., 2017). Although LVAs are often targeted during ablation therapy, there is no standardized methodology for defining them, and appropriate voltage thresholds still require histological validation.

##### Omnipolar mapping

**Figure 6:**
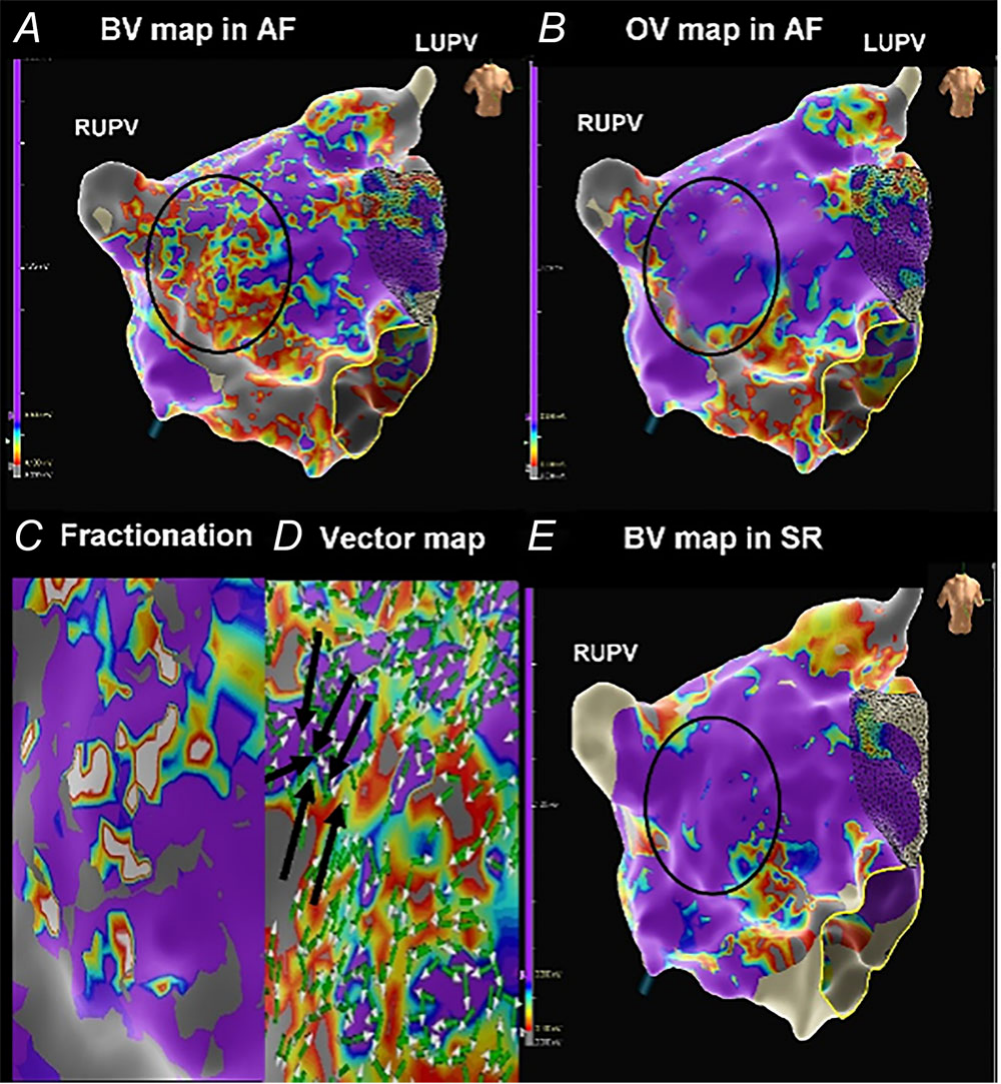
Voltage mapping Illustration of different voltage mapping. BV map of the LA in AF (*A*) and OV map of the LA in AF (*B*), both shown from an anterior–posterior view, indicating differences in low voltage area distribution. The BV map highlights extensive low voltage in the anterior wall, which is less pronounced in the OV map. The LVA presented on the BV map (absent on OV map) corresponds to a site of wavefront collision as shown in the fractionation map (*C*) and the activation vector map (*D*). *E*, the BV map in SR aligns more closely with the OV map in AF that with the BV map in AF. BV, bipolar voltage; OV, omnipolar voltage; LUPV, left upper pulmonary vein; RUPV, right upper pulmonary vein; RLPV, right lower pulmonary vein (Butcher et al., 2023).

Omnipolar mapping addresses the wavefront direction dependence seen in bipolar mapping by providing orientation independent voltage measurements (Haldar et al., 2017; Massé et al., 2016). A study by Haldar et al. (2017) demonstrated that scar maps in SR and AF varied with the orientation of bipoles, but omnipolar mapping eliminated this dependence by extracting maximal voltage independent of direction, wavefront collision, or fractionation during AF. Additionally, omnipolar mapping of voltage in AF was found to better correlate with voltage mapping in SR overcoming the effects of fractionation and wavefront collision (Butcher et al., 2023) (Fig. 6). It also showed improved accuracy in identifying PV reconnection sites in AF compared to bipolar mapping. This makes omnipolar mapping a valuable tool for more precise assessment and PV reconnection site identification in AF.

Although omnipolar mapping offers many advantages, it also has its limitations. Deno et al. (2017) highlighted a key assumption in omnipolar mapping, such that the catheter is positioned on a flat myocardium. However, this assumption may not hold in curved or trabeculated areas, potentially affecting catheter orientation and accuracy. Additionally, omnipolar mapping faces challenges related to algorithm implementation. Some systems restrict its use to specific proprietary catheters or software, and assumptions about wavefront propagation may not always be valid in complex scarred cardiac tissue (Dittrich et al., 2024; Karatela et al., 2022). Although omnipolar mapping can be applied offline using EGMs from any mapping system, EnsiteX (Abbott, Chicago, IL, USA) is currently the only system integrating it in real time. Moreover, its implementation in EnsiteX depends on the HD Grid Catheter making it incompatible with other catheter types.

#### CV mapping

**Figure 7:**
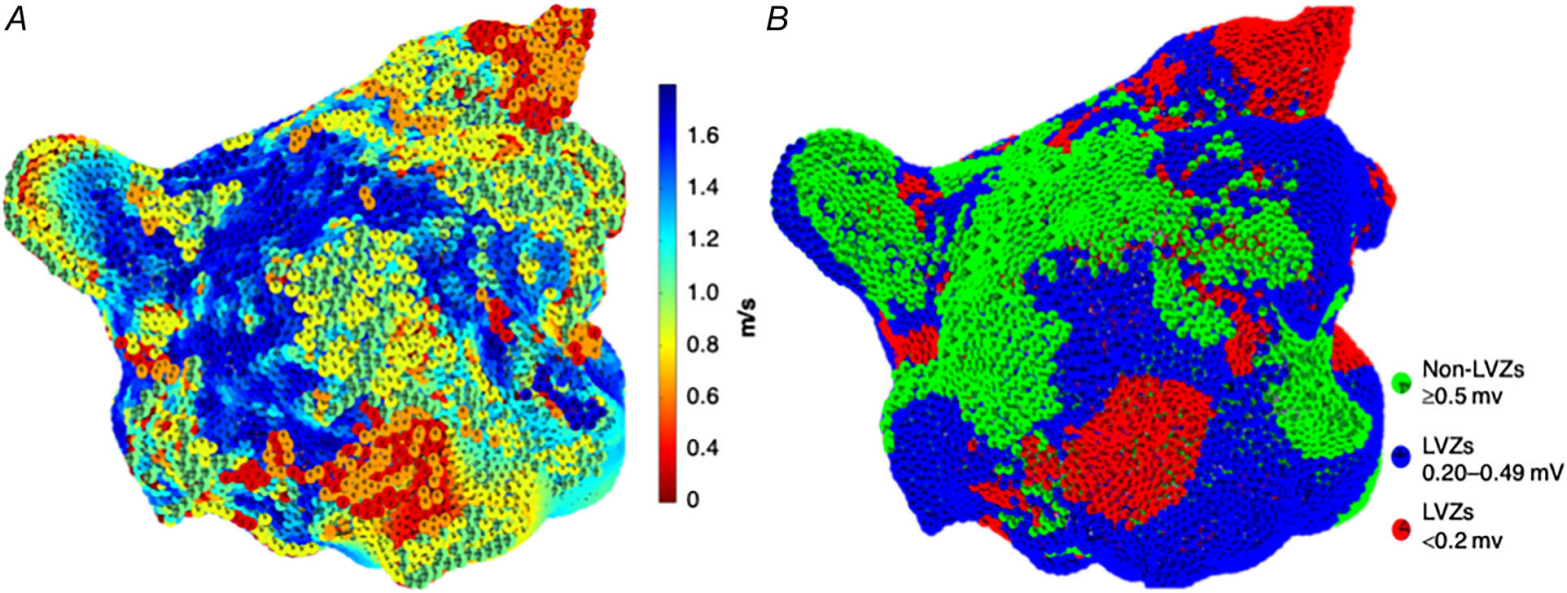
CV and BV mapping Illustration of CV mapping and BV mapping. *A*, CV map demonstrating the relationship between CV and voltage. *B*, BV map, non‐LVAs represented by green dots, LVAs (0.20–0.49 mV) represented by blue dots, and very LVAs represented by red dots (Honarbakhsh, Roney et al., 2024).

CV measures the speed and direction of electrical wavefront propagation through the myocardium and reflects substrate health. CV mapping, which is influenced by factors including   fibrosis, fibre alignment, cell‐to‐cell coupling and sodium current changes, can help identify potential ablation targets. Regions of low CV are often associated with fibrosis and are critical for sustaining re‐entry circuits in AF (Fig. 7). Mapping atrial CV provides crucial insights into arrhythmia mechanisms, helping to identify drivers, predict re‐entrant pathways and personalize treatment strategies.

CV exhibits rate dependence, known as restitution, where conduction velocity changes with pacing rates. Dynamic CV assessments across different pacing intervals reveal that healthy tissue exhibits higher CV values than LVAs, with moderate fibrosis showing a steady CV decrease at higher rates, whereas scarred regions demonstrate minimal change (Honarbakhsh et al., 2019a; Honarbakhsh et al., 2019b; Honarbakhsh, Horrach et al., 2024). Rate‐dependent CV (RDCV) slowing sites predominantly co‐localise with moderate LVAs rather than fixed scar, highlighting that not all LVAs contribute equally to AF (Honarbakhsh et al., 2019b; Honarbakhsh, Roney et al., 2024). Functional remodelling plays an important role because RDCV slowing sites are more frequent in areas with both fixed and functional remodelling than in those with only fixed remodelling. These distinctions underscore the mechanistic significance of RDCV dynamics in arrhythmogenesis (Lalani et al., 2012; Markides et al., 2003) and suggest RDCV slowing as a potential novel ablation target, highlighting the need for further research to confirm its efficacy and improve LVA stratification based on mechanistic relevance in sustaining AF (Honarbakhsh, Roney et al., 2024).

The combination of unipolar, bipolar and omnipolar mapping modalities to map CV and LVAs may better capture different aspects of atrial remodelling. For example, the observation of high CVs and large unipolar voltages in areas of low omnipolar voltage by van Schie et al. (2021) suggests that the combination of low unipolar and low omnipolar voltage may provide a more accurate indication of true LVAs indicating fibrosis (Haldar et al., 2017).

### Blood biomarkers of atrial fibrosis

A detailed review of blood biomarkers of atrial fibrosis is presented in Schotten et al. (2025). Here, we provide a brief overview of the main blood biomarkers used to detect atrial fibrosis in patients with AF. These blood biomarkers include C‐reactive protein (CRP), N‐terminal‐pro‐B‐type natriuretic peptide (NT‐proBNP), and high‐sensitivity troponin I (Hijazi et al., 2013). These biomarkers are often elevated in AF patients and are correlated with inflammatory processes and cardiac stress that contribute atrial fibrosis. Another blood biomarker used is interleukin‐6 (IL‐6), which is a pro‐inflammatory cytokine that is often elevated in patients with AF, contributing to the development and progression of fibrosis in patients with AF (Ma et al., 2017). CRP and IL‐6 are key inflammatory markers linked to fibrosis development, and NT‐proBNP and high‐sensitivity troponin I reflect cardiac stress, often being elevated as a result of the increased workload on the atria (Blake & Ridker, 2003; Boos et al., 2006; Hijazi et al., 2013; Velt et al., 2024). Although they cannot provide detailed information on the spatial distribution of atrial fibrosis, tracking fibrosis‐related biomarkers can help in risk assessment for AF patients, helping to identify those with increased risk for AF, AF progression and other complications such as stroke (Hijazi et al., 2013). Recent research demonstrated that increased levels of serum bone morphogenic protein 10 (BMP10) were associated with persistent AF history (Winters et al., 2024), as well as providing a marker for increased risk of ischaemic stroke and mortality (Hennings et al., 2023; Hijazi et al., 2023). Winters et al. (2024) also showed that increased levels of BMP10 were correlated with higher incidences of late postoperative AF and left atrial appendage endomysial fibrosis. However further research on the role of BMP10 in atrial fibrosis and its effect regulating cellular mechanisms is needed to further mechanistic understanding.

## Impact of fibrosis in treatment outcomes

Atrial fibrosis affects electrical and structural remodelling, contributing to the AF substrate. As such, treatments may aim to limit the progression of atrial fibrosis or may target areas of atrial fibrosis as critical regions for initiating and sustaining AF. There are various approaches that are used to limit fibrosis progression in AF, and therefore limit possible development and/or progression of AF, by targeting molecular pathways (Fang et al., 2017). A detailed review of therapeutic approaches for treatment of atrial fibrosis including anti‐fibrotic drug therapies is provided in Schotten et al. (2025). This section reviews how catheter ablation therapy approaches may target potential regions of atrial fibrosis identified through LGE‐CMR or using EAM voltage mapping.

### Low‐voltage guided ablation and LGE‐CMR guided catheter ablation approaches

LVAs are often associated with atrial fibrosis, as fibrotic tissue contributes to the creation of non‐conductive or poorly conductive areas within the atria, which underlie the development of LVAs and their role in arrhythmogenesis (Starek et al., 2023). Research has indicated that re‐entry rotations, which are linked to AF, often to occur in LVAs, and the burden of LVAs correlates with the burden of re‐entry rotations (Honarbakhsh, Schilling, Dhillon et al., 2018; Honarbakhsh, Schilling, Providencia et al., 2018; Honarbakhsh et al., 2022). As a result, LVAs are increasingly being targeted in AF ablation strategies, such as Box isolation of fibrotic areas (i.e. BIFA), which creates patient‐specific lesions around low‐voltage tissue (Kottkamp et al., 2016). Clinical studies, such as that by Jadidi et al. (2016) have shown improved outcomes with combined PVI and voltage‐guided ablation for persistent AF. A meta‐analysis also supports the efficacy of ablation of LVAs for improving AF outcomes (Khurram et al., 2014). Furthermore, the ERASE AF trial suggested that patients with LVAs who underwent PVI plus substrate modification had higher freedom from AF/AT than those who only received PVI, highlighting the benefit of substrate modification in these patients (Huo et al., 2022).

The efficacy of low‐voltage ablation appears to depend on anatomical characteristics. In the SUPPRESS‐AF trial, a multicentre study of 343 patients with persistent AF, no significant difference in AF recurrence was found between those receiving low‐voltage ablation combined with PVI and those receiving PVI alone. However, a subgroup of patients with left atrial enlargement saw a 40% reduction in arrhythmia recurrence with low‐voltage ablation (Huo et al., 2022). Substrate modification of LVAs has also been studied in PAF, with the VOLCANO trial showing no improvement in AF recurrence‐free survival when LVA ablation was added to PVI in PAF patients, and a higher rate of atrial tachycardia in the long term (Masuda et al., 2020). By contrast, another study of older patients (65–80 years) with PAF found that LVA ablation plus PVI resulted in a lower rate of atrial arrhythmia recurrence compared to PVI alone (Chen et al., 2023).

As an alternative to EAM LVA ablation, LGE‐CMR may be used to guide ablation of regions of atrial fibrosis. For example, the DECAAF‐II trial used delayed‐enhancement MRI to guide fibrosis‐targeted ablation, finding no added benefit in targeting fibrotic areas identified by LGE compared to conventional PVI alone for reducing AF recurrence rates (Bisbal et al., 2020; Marrouche et al., 2022). These techniques aim not only to manage AF, but also to reduce the structural changes associated with fibrosis.

Alternative ablation strategies using LGE‐CMR include targeting fragmented fibrosis, fibrosis homogenisation and ‘dechannelling’ fibrotic isthmi. Ablating fragmented LGE areas, hypothesised to anchor meandering AF drivers, showed promise in a study of 31 patients (Kiuchi et al., 2021). Similarly, fibrosis homogenisation and dechannelling were used for patients with recurrent atrial arrhythmias, with comparable outcomes between LGE‐CMR‐guided and conventional methods (Fochler et al., 2019). However, the overall efficacy of these approaches requires further investigation, and future studies should refine the optimal imaging features for guiding ablation.

## Digital twins: personalised cardiac modelling

Digital twins in healthcare represent a virtual framework that dynamically integrate clinical data using mechanistic and statistical models. A fully developed digital twin model incorporates both population‐level and patient‐specific representations for optimal clinical recommendations (Corral‐Acero et al., 2020). In cardiac electrophysiology, digital twin models enhance understanding at cellular, tissue and organ levels, revealing mechanisms that cannot be detected by conventional clinical techniques (Bhagirath et al., 2024; Niederer et al., 2019) (Fig. 9). Advances in computational modelling allow for patient‐specific simulations using EAM and/or imaging data for AF, can aid predictive diagnosis, treatment (Azzolin et al., 2023; Bhagirath et al., 2024; Boyle et al., 2019; Roney et al., 2023). By incorporating patient‐specific anatomy and fibrosis, these models facilitate mechanistic research of AF and fibrosis. Varying physiological parameters enables researchers to assess their impact on AF and identify mechanistic causes. Additionally, digital twins allow *in silico* testing of different treatment strategies, optimizing therapy selection before applying it to clinical settings. Large‐scale patient‐specific or artificial cohorts support the evaluation of new treatments in cost‐effective, low‐risk *in silico* clinical trials (Clayton, 2018; Roney et al., 2022; Sun et al., 2023).

**Figure 8:**
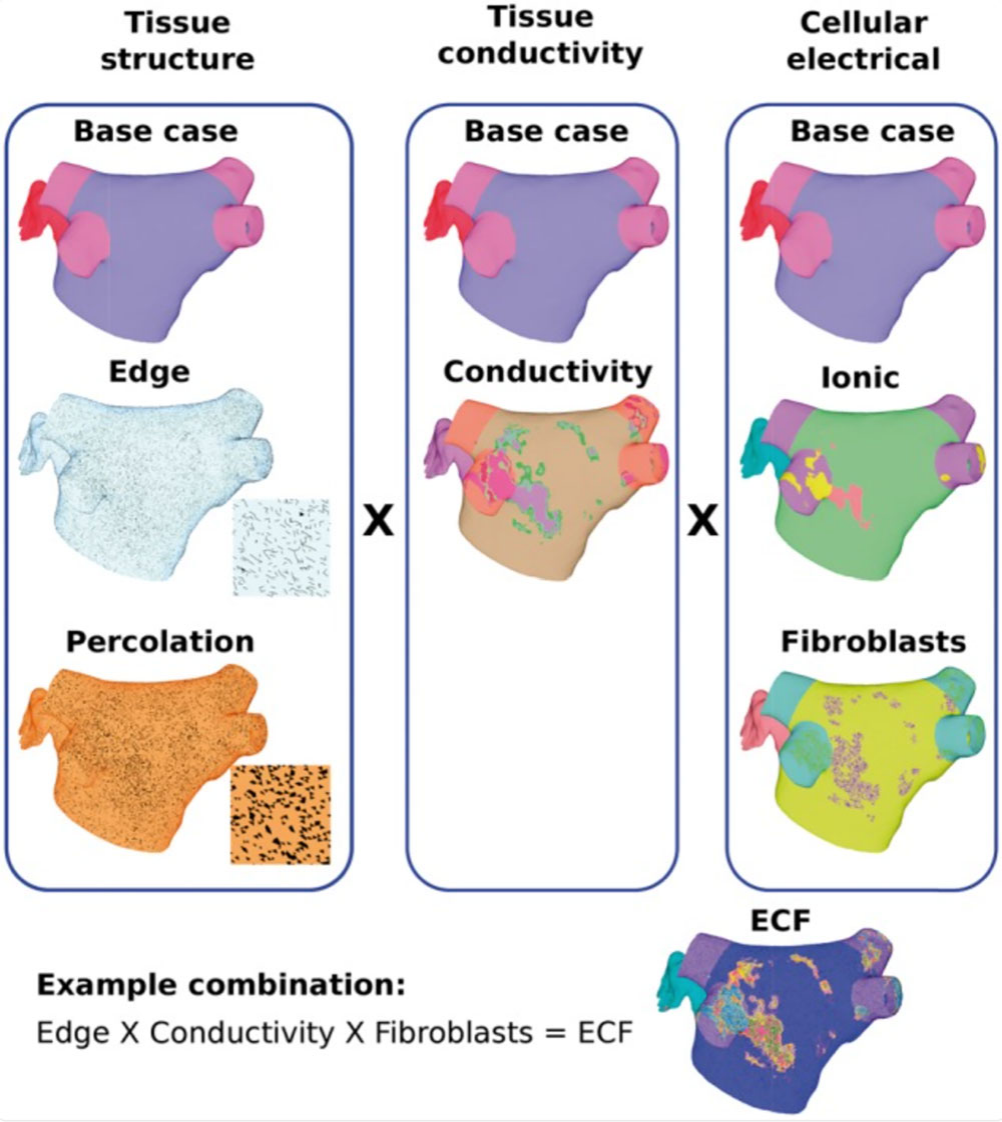
Different fibrosis models Different fibrosis models combining tissue structure {none, edge, percolation}, conductivity {none, conductivity} and cellular electrical changes {none, ionic, fibroblasts}. In the edge mesh, black lines mark split edges and black regions indicate removed faces similar to the percolation mesh. One of 18 combinations, ECF (edge, conductivity fibroblasts), is shown below. Colours in C, F, I and ECF meshes denote regions with different conductivity, ionic properties or cell models (Roney et al., 2016). E, Edge; P, Percolation; C, Conductivity; I, Ionic; F, Fibroblasts.

The inclusion of fibrosis is a key area in patient‐specific AF modelling. Various techniques have been used to integrate fibrosis information into these models; for example, by including structural changes such as interstitial conduction barriers and probabilistic percolation, altered conductivity and changes in cellular electrical properties through fibroblast incorporation, as well as ionic channel modifications (Roney et al., 2016) (Fig. 8).

**Figure 9:**
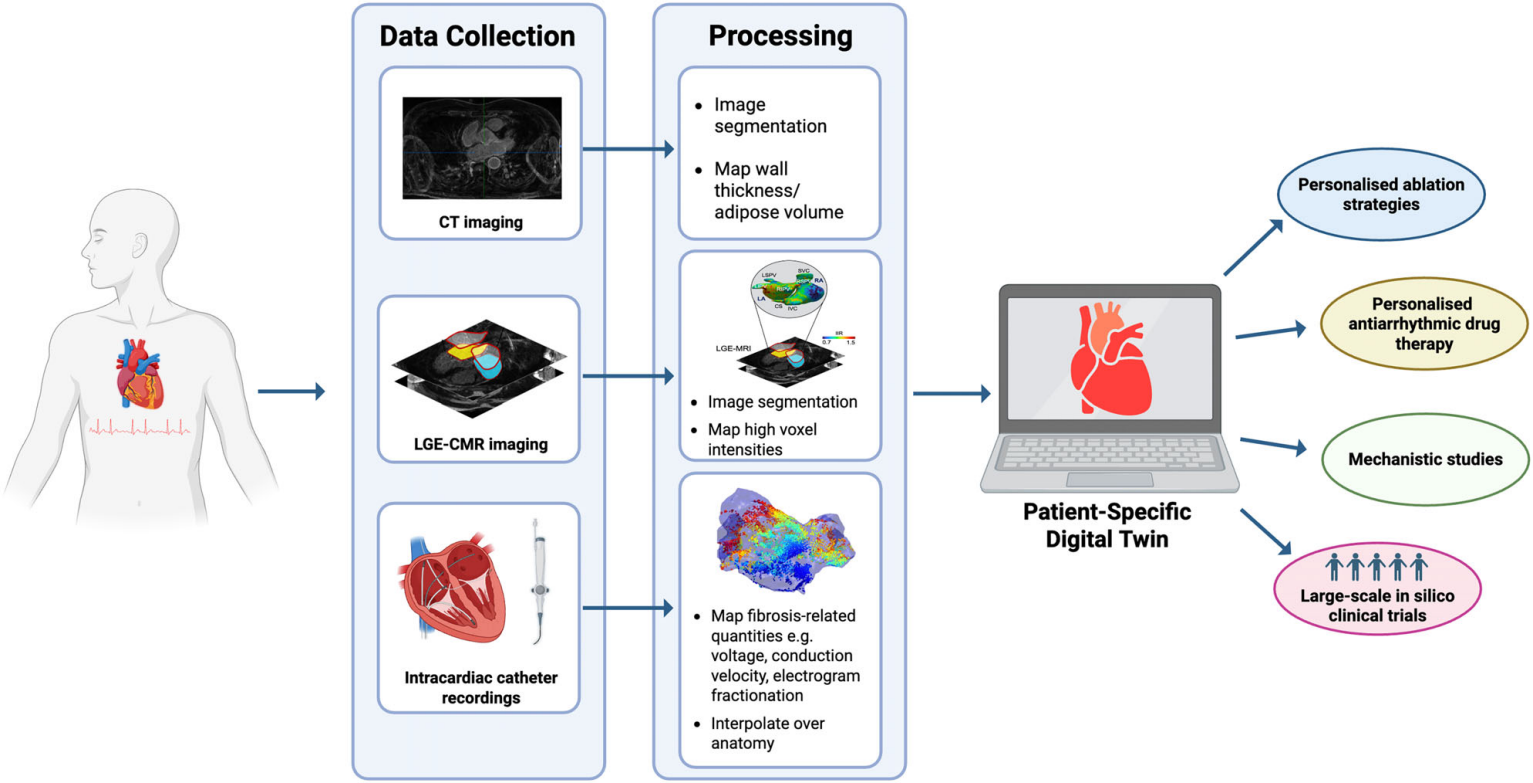
Cardiac digital twins for atrial fibrillation Overview of cardiac digital twins for AF, incorporating patient‐specific anatomical and fibrosis information from clinical data. An overview is provided of the main methods of fibrosis detection and mapping for personalized electrophysiological modelling and potential uses of cardiac digital twins.

Several studies have incorporated the effects of replacement fibrosis or interstitial fibrosis on wavefront propagation and electrogram. In volumetric models, fibrosis is often increased on the epicardium, and atrial fibrosis is more pronounced in the subepicardial layers than in the endocardial muscle bundles (Masè et al., 2024; Vigmond et al., 2016). Balaban et al. (2020) developed a computational method to simulate life‐threatening arrhythmias in non‐ischaemic cardiomyopathy by modelling interstitial fibrosis and replacement fibrosis aiming to investigate how the modelling methodology for fibrosis influences arrhythmia mechanisms (Balaban et al., 2020; Myklebust et al., 2024). Furthermore, the effect of fibroblast coupling to myocytes can be modelled using several fibroblast cell models with different fibroblast densities resulting in different electronic effects (Colman et al., 2023; Jacquemet & Henriquez, 2007; Sánchez et al., 2019). Fibrosis can also be modelled at the cellular level by modifying ionic conductance to represent its effect on electrophysiology (Zahid et al., 2016). Additionally, the models incorporating ionic changes in fibrotic remodelling can also be combined with conductivity changes. For example, in the modelling methodology of the OPTIMA trial, conductivity values are reduced with alterations in the anisotropy ratio, representing interstitial fibrosis, gap junction remodelling and higher disruption of cell‐to‐cell coupling in the transverse direction of cardiac fibres, together with modifications at the cellular level. A review by Sánchez and Loewe 2022 emphasized how multiscale models facilitate the analysis of the different mechanisms driving complex fractionated atrial electrograms and the detection of fibrosis in electrograms.

Several studies have investigated the impact of fibrosis quantification and modelling on simulated fibrillatory dynamics in computational models of AF. Honarbakhsh, Horrach et al. (2024) investigated the effect of fibrosis modelling on rotational driver burden in AF. Fibrosis based on peak‐to‐peak bipolar voltage values was incorporated using several different methods, including scaling and conductivities and replacement fibrosis through probabilistic percolation. PVI was simulated to assess rotational driver burden, providing insight of the impacts slow CV and scar have on rotational driver burden into fibrosis‐driven AF mechanisms (Honarbakhsh, Horrach et al., 2024). Roney et al. (2016) implemented several different methods of modelling fibrosis in patient‐specific AF models in patients with non‐invasive electrocardiographic imaging recordings of AF episodes were available, comparing rotational activity dynamics and locations to the clinically measured rotational activity locations. Fibrotic regions were modelled using structural changes, with interstitial fibrosis represented by non‐conducting barriers along the edges of the mesh in the longitudinal fibre direction and replacement fibrosis represented by probabilistic percolation, as well as lowered conductivity, and cellular electrical alterations represented by ionic modification, as well as coupling to fibroblasts. These were assessed individually or in combination to better understand the different fibrotic effects in AF dynamics, showing that the way fibrosis is represented in AF models significantly impacts rotor dynamics. This must be carefully taken into account for patient‐specific modelling (Roney et al., 2016).

The use of digital twins in personalized cardiac modelling possesses many advantages as explained above. However, there are some limitations and areas of uncertainty when developing these models. Some personalised cardiac models focus on specific aspects of cardiac tissue physics that make it challenging to represent the complex arrhythmogenic substrates, such as modelling fibrosis or areas of necrotic infarcts (Connolly & Bishop, 2016; Niederer et al., 2019; Sánchez & Loewe, 2022). Parameter sensitivity can lead to significant prediction difference, and developing accurate parametrization can be challenging (Deng et al., 2017). Additionally, some models lack the spatial and temporal resolution needed to capture 3‐D dynamics (Lopez‐Perez et al., 2019). Validation remains a challenge as a result of the limited experimental data available, and model credibility assessment methods are still evolving (Galappaththige et al., 2022). Computational intensity is also a limitation because high‐resolution simulations require significant computational resources, but reduced‐order or simplified eikonal models have been proposed to improve feasibility (Alberto Barrios Espinosa et al., 2022). Uncertainty in EAM data collection and model equations can limit accuracy, especially when extrapolating different conditions (Clayton et al., 2020; Wallman et al., 2014). Furthermore, the current models do not account for interactions with other pathological conditions. Lack of knowledge of cardiac electrophysiology, including mechanisms such as fibroblast coupling and mechano‐transduction, further restrict model precision (Trayanova et al., 2024). Patient‐specific variability adds another layer of complexity because general models may not fully represent individual differences, encouraging the development of population‐based virtual cohorts (Niederer et al., 2020; Roney et al., 2022). Finally, ethical and practical challenges, including the need for accurate clinical data and regulatory approval, must be addressed before these models can be widely implemented.

## Future directions

This section highlights recent advancements in and potential future directions for mapping techniques, fibrosis quantification and personalized cardiac modelling, which, although not yet integrated into routine clinical practice, have the potential to enhance our approach to ablation therapy and improve outcomes for patients with AF.

### Advancements in biomarkers and early detection

Early detection of atrial fibrosis is crucial for improving treatment outcomes in AF. Early detection of atrial fibrosis is crucial for improving treatment outcomes in patients with AF.  Current diagnostics approaches rely strongly on imaging modalities such as LGE‐CMR, which are effective but may not always be accessible or feasible for routine clinical use (Zhang et al., 2024). As an alternative approach, there is current interest on biomarkers that are involved in extracellular matrix remodelling, inflammation, and fibrotic activity (Angeli et al., 2024; Schotten et al., 2025). Such biomarkers have been explored for their association with atrial fibrosis in patients with AF (see section on ‘Blood biomarkers of atrial fibrosis’). These biomarkers, when combined with clinical parameters, can provide a minimally invasive means of risk stratification and early detection of fibrotic remodelling (Begg et al., 2020; Ding et al., 2020; Koniari et al., 2021). AI‐driven models involving biomarker data with imaging modalities could be used as potential tools to predict the risk of AF in patients.

Machine learning techniques have been used to develop multimodal predictive models incorporating electrocardiographic signatures of atrial fibrosis, such as p‐wave indices and fragmentation, with clinical and imaging data to improve AF risk stratification (Filos et al., 2017; Luongo et al., 2021; Razeghi et al., 2020; Sánchez, Luongo et al., 2021). Future research should focus on validating these approaches on large‐scale datasets, and also assess their utility in guiding personalized therapy. Additionally, further research is needed to evaluate the dynamic alterations in these biomarkers over time and their association with fibrosis progression, which could further enhance early detection and provide better treatment approaches for patients with AF.

### Identifying endomysial fibrosis

The type and location of fibrosis significantly influence AF properties, with animal studies suggesting that endomysial fibrosis, comprising fibrous tissue within individual myocytes, rather than perimysial fibrosis surrounding myocyte bundles, increases the complexity of fibrillatory conduction (Verheule et al., 2013). Maesen et al. (2022) demonstrated that endomysial fibrosis content, rather than overall fibrosis, determines AF complexity, linking increased myocyte‐to‐myocyte distance in the right atrial appendage to a higher number of wavefronts, breakthroughs and fractionation index. Because endomysial fibrosis cannot be measured with LGE‐CMR, new methodologies and metrics are needed to better assess atrial fibrosis properties relevant to AF mechanisms. Future research should also consider how patient demographics such as age, biological sex and lifestyle influence atrial remodelling and AF progression in the interpretation of imaging and electroanatomic data.

### Improved monitoring of structural remodelling

Assessing and tracking atrial fibrosis progression over time remains a significant challenge in AF management. Although LGE‐CMR is an essential technique for visualizing atrial fibrosis, it has its limitations. To overcome these limitations, advanced imaging modalities need to be explored, which offer high‐resolution images and a more detailed assessment of atrial structural remodelling. Higher resolution MRI techniques that allow higher spatial resolution and faster acquisition times, as well as detailed representation of atrial tissue, could potentially improve detection and quantification of fibrosis. Additionally, research into new contrast agents that enhance the contrast‐to‐noise ratio and specificity of atrial fibrosis can further improve the precision of LGE‐CMR (Cochet et al., 2018; Luetkens et al., 2018; Siebermair et al., 2017; Zghaib & Nazarian, 2018). Advanced non‐invasive techniques, such as myocardial T1 mapping, show promise for evaluating myocardial fibrosis. This technique allows for a safer non‐invasive assessment of the dynamics of fibrosis in myocardial atrial tissue, without the use of contrast agents, further enhancing atrial fibrosis detection and management in patients with AF (Moon et al., 2013; Taylor et al., 2016).

### *In silico* trials to inform ablation therapy

Personalised approaches in managing AF are critical to improve prediction and potentially reduce mortality rates. One promising development is the use of personalized cardiac digital twins to improve AF treatment. This concept, involving biophysical simulations and statistical models, has shown potential for advancing catheter ablation therapy. For example, the ongoing OPTIMA trial at Johns Hopkins University is comparing PVI alone *vs*. PVI combined with targets identified through biophysical simulations in patients with persistent AF and atrial fibrotic remodelling (Boyle et al., 2019). Early results suggest that simulations based on LGE‐CMR data can help identify fibrotic regions sustaining AF, offering a promising approach for patients with significant fibrotic remodelling (Boyle et al., 2019).

Further research can explore the use of virtual population trials to assess ablation or antiarrhythmic drug therapies. For example, Roney et al. (2021, 2022) showed how ablation outcomes varied depending on anatomical properties and fibrotic remodelling. In another example, Zolotarev et al. (2024) utilised 1000 Statistical Shape Models of biatrial anatomies to predict the acute ablation outcome based on feature maps calculated from AF biophysical simulation before ablation. The study showed that strategies with ablation of areas with high fibrosis significantly outperformed efficacy (Zolotarev et al., 2024). Future directions for patient‐specific biophysical modelling include refining uncertainty quantification methodologies for rapid calibration tor patient‐specific data (Martínez Díaz et al., 2024) and combining EAM with fast simulation techniques to personalize these biophysical simulations. This research is expected to enhance the precision of AF treatments and improve patient outcomes (Williams et al., 2019). Other future directions include integrating deep learning‐based decision support systems into clinical practice, which has the potential to optimize AF management and prevent AF recurrence.

Fibrosis distributions affect the signal propagation through the heart tissue and can be utilized to create a simple *in silico* playground in large populational *in silico* trials for predicting the best ablation strategy. The lack of clinical data (LGE‐CMR scans) and concerns about its privacy raise a motivation for the creation of clinically and physiologically relevant synthetic fibrosis distributions. There are several examples of successful implementation of different statistical deep learning techniques for this goal (Clayton, 2018; Lawson et al., 2024; Zolotarev et al., 2025). Synthetic datasets can help overcome data scarcity and bias, enabling more reliable training of prediction pipelines. Developing predictive tools based on deep learning models and synthetic datasets can enhance diagnostic accuracy and personalize treatment plans for AF, potentially leading to improved patient outcomes.

## Conclusions

The extent of fibrosis is a key indicator of AF progression, with persistent AF patients exhibiting both atrial fibrosis and AF‐related remodelling. Multiple modalities can be used to assess atrial fibrosis, including imaging, electroanatomic mapping and blood biomarkers, each utilising different characteristics of fibrotic tissue to define fibrotic regions and with different limitations. Electroanatomic mapping methods show promise, helping to identify regions of structural and functional remodelling, with measurements influenced by electrogram type (unipolar, bipolar or omnipolar), mapping rhythm and assessment metrics (voltage amplitude or conduction velocity). Fibrosis, conduction velocity heterogeneity and functional remodelling detected by EAM affect wavefront propagation in sinus rhythm and the burden of rotational activity in AF. Such metrics provide valuable insights into AF and atrial fibrosis pathophysiology and show potential for identifying novel treatment targets. The integration of fibrosis mapping in personalised computational models, and utilisation of large‐scale *in silico* clinical trials and machine learning approaches can improve prediction timescales for realistic clinical use; however, further study is required the determine standardised approaches to detect and model atrial remodelling and different types of fibrosis from clinical modalities. As we move closer towards Digital Twins in Healthcare, the consideration of such physiological factors, as well as continued collaboration between clinical and modelling teams, remains crucial (EDITH consortium, 2023).

## Additional information

### Competing interests

The authors declare that they have no competing interests.

### Author contributions

C.V.H. and C.H.R. conceptualized the review. C.V.H., C.H.R., L.B., C.N., M.E. and A.Z. contributed to writing. C.V.H., C.H.R. and L.B. contributed to editing. C.V.H., L.B., C.N. and S.B.M. contributed to preparation of figures. Figures were generated using Biorender. S.A.A., S.H. and C.H.R. contributed to the design of the review and data presentation. All listed authors contributed to the work and read and approved the final version of the manuscript submitted for publication.

### Funding

This research was funded by a UKRI Future Leaders Fellowship (MR/W004720/1). Dr Laura Bevis funding – the authors (LB specifically) acknowledge funding from Queen Mary University of London in contribution towards the Digital Europe Programme of the European Commission (grant agreement 101083771) for the EDITH project. We also acknowledge funding from the National Institute for Health Research Barts Biomedical Research Centre (NIHR203330). Dr Shohreh Honarbakhsh funding – this study was funded by the British Heart Foundation Clinical Intermediate Fellowship FS/ICRF/22/26034.

## Supporting information


Peer Review History
