# Supplementary material for: Atrial fibrosis in atrial fibrillation: Mechanisms, mapping techniques and clinical applications
Source: J Physiol. 2025 May 28;604(13):5103–25. doi: 10.1113/JP288680 (PMC13327766; doi:10.1113/JP288680)
Supplement: Supplementary file 1 — Peer Review History [file TJP-604-5103-s001.pdf]

The Journal of Physiology

Atrial Fibrosis in Atrial Fibrillation: Mechanisms, Mapping Techniques, and Clinical Applications

Caterina Vidal Horrach, Laura Bevis, Cynthia Nwanna, Alexander Zolotarev, Mahmoud Ehnesh, Semhar Biniam Misghina, Sayed Al-Aidarous, Shohreh Honarbakhsh, and Caroline H Roney
DOI: 10.1113/JP288680

Corresponding author(s): Caroline Roney (c.roney@qmul.ac.uk)

The following individual(s) involved in review of this submission have agreed to reveal their identity: Stef Zeemering (Referee #1); Miguel Rodrigo (Referee #2)

Review Timeline:

Submission Date:	31-Jan-2025
Editorial Decision:	21-Feb-2025
Revision Received:	21-Mar-2025
Accepted:	08-Apr-2025

Senior Editor: Bjorn Knollmann

Reviewing Editor: Axel Loewe

Transaction Report:

(Note: With the exception of the correction of typographical or spelling errors that could be a source of ambiguity, letters and reports are not edited. Depending on transfer agreements, referee reports obtained elsewhere may or may not be included in this compilation. Referee reports are anonymous unless the Referee chooses to sign their reports.)

Dear Dr Roney,

Re: JP-TR-2025-288680 "Mapping Techniques for Evaluating Fibrosis in Patients with Atrial Fibrillation" by Caterina Vidal Horrach, Laura Bevis, Cynthia Nwanna, Mahmoud Ehnesh, Semhar Biniam Misghina, Sayed Al-Aidarous, Shohreh Honarbakhsh, and Caroline H Roney

Thank you for submitting your manuscript to The Journal of Physiology. It has been assessed by a Reviewing Editor and by 2 expert referees and we are pleased to tell you that it is acceptable for publication following satisfactory revision.

The referee reports are copied at the end of this email.

Please address all the points raised and incorporate all requested revisions or explain in your Response to Referees why a change has not been made. We hope you will find the comments helpful and that you will be able to return your revised manuscript within 4 weeks. If you require longer than this, please contact journal staff: jp@physoc.org.

Your revised manuscript should be submitted online using the link in your Author Tasks: Link Not Available. This link is accessible via your account as Corresponding Author; it is not available to your co-authors. If this presents a problem, please contact journal staff (jp@physoc.org). Image files from the previous version are retained on the system. Please ensure you replace or remove any files that are being revised.

If you do not wish to submit a revised version of your manuscript, you must inform our journal staff (jp@physoc.org) or reply to this email to request withdrawal. Please note that a manuscript must be formally withdrawn from the peer review process at one journal before it may be submitted to another journal.

TRANSPARENT PEER REVIEW POLICY: To improve the transparency of its peer review process, The Journal of Physiology publishes online as supporting information the peer review history of all articles accepted for publication. Readers will have access to decision letters, including Editors' comments and referee reports, for each version of the manuscript, as well as any author responses to peer review comments. Referees can decide whether or not they wish to be named on the peer review history document.

ABSTRACT FIGURES: Authors may use The Journal's premium BioRender account to create/redraw their Abstract Figures (and any other suitable schematic figure). Information on how to access this account is here: https://physoc.onlinelibrary.wiley.com/journal/14697793/biorender-access.

This will enable Authors to create and download high-resolution figures. If authors have used the free BioRender service, they can use the instructions provided in the link above to download a high-resolution version suitable for publication. The link provided should only be used for the purposes of this submission. Authors will be charged for figures created on this account if they are not related to this manuscript submission.

LANGUAGE EDITING AND SUPPORT FOR PUBLICATION: If you would like help with English language editing, or other article preparation support, Wiley Editing Services offers expert help, including English Language Editing, as well as translation, manuscript formatting, and figure formatting at www.wileyauthors.com/eeo/preparation. You can also find resources for Preparing Your Article for general guidance about writing and preparing your manuscript at www.wileyauthors.com/eeo/prepresources.

REVISION CHECKLIST: Upload a full Response to Referees file. To create your 'Response to Referees' copy all the reports, including any comments from the Senior and Reviewing Editors, into a Microsoft Word, or similar, file and respond to each point, using font or background colour to distinguish comments and responses and upload as the required file type.

Please upload two versions of your manuscript text: one with all relevant changes highlighted and one clean version with no changes tracked. The manuscript file should include all tables and figure legends, but each figure/graph should be uploaded as separate, high-resolution files.

You may also upload:

- 'Potential Cover Art' for consideration as the issue's cover image
- Appropriate Supporting Information (Video, audio or data set: see https://jp.msubmit.net/cgi-bin/main.plex?form_type=display_requirements#supp).

We look forward to receiving your revised submission.

If you have any queries, please reply to this email and we will be pleased to advise.

Yours sincerely,

Bjorn Knollmann

Senior Editor
The Journal of Physiology

EDITOR COMMENTS

Reviewing Editor:

Your manuscript was reviewed by two expert referees who acknowledge that this is a relevant and well-developed review. In their reports, both referees suggest to further sharpen the focus of this review, which I support. The final focus should also be reflected in the title.

The recent consensus statement on atrial cardiomyopathy seems another reference worthwhile mentioning: https://dx.doi.org/10.1093/europace/euae204

Please also see 'Required Items' below.

Senior Editor:

I concur with the reviewing editor's assessment.

REFEREE COMMENTS

Referee #1:

The authors provide a broad overview of the many aspects that play a role in the assessment and impact of atrial fibrosis, from mechanisms that lead to (different types of) fibrosis, techniques to quantify fibrosis (non)-invasively, the treatment options with respect to fibrosis (prevention, ablation,...), and computational tools that incorporate fibrosis to personalise treatment (digital twins) and to perform in-silico trials that consider structural remodelling.

Review

In general, the manuscript is well-written, and well-structured. The title (Mapping Techniques for Evaluating Fibrosis in Patients with Atrial Fibrillation) suggests that the review is primarily about techniques to assess the presence and degree of fibrosis in patients. The main text however also deals with several other aspects (mechanisms, treatment, computational modelling) of atrial fibrosis in detail. Perhaps a different title will better cover the content. Alternatively, one could consider shortening the sections on mechanisms and therapy not related to mapping of fibrosis, and focus more on mapping and computational modelling.

Specific remarks

1. Role of fibrosis

Arrhythmogenic Effects of Fibrotic Remodelling: consider to discuss the role of fibrosis in the development of endo-epicardial dissociation (see for instance doi: 10.1161/CIRCEP.113.000342)

2. Mapping techniques

- Section 2.3.1: in lines 335 the authors paint a bleak picture of using LGE-CMR to quantify atrial fibrosis, by listing all the factors that may affect the quantification. Are there any efforts to standardize the methodology, apart from automated segmentation, and move the field forward?

- Section 2.4.1.3: Omnipolar mapping: are there any limitations for this technique that are worth mentioning, such as dependence on catheter configuration / proprietary algorithms?

- Section 2.5: here, the atrial-specific blood biomarker BMP10 is missing from the discussion (see for instance doi: 10.1016/j.jacep.2024.03.003)

4. Digital twins

- This section would benefit from better structuring, better balance between general description and details.

- Address limitations of digital twin modelling and how to mitigate/disseminate model uncertainty.

5. Future directions

The discussion here is rather one-sided, focusing mostly on a selection of computational approaches/tools that are currently developed or tested. Other topics could be the development of new diagnostic markers, early detection, and improved monitoring of progression/regression of structural remodeling.

Referee #2:

The authors present an interesting review focusing on an important topic in the field of atrial electrophysiology. They make a great review of atrial fribrosis, covering from the physiological point of view to its application in computational models. The article is very interesting for an audience focused on AF mapping and/or computational modelling, which is welcomed for those working in this field as it nicely links the patho-phisiology of fibrosis infiltration with mapping and computational models.

However, reading it gives the feeling that the authors have tried to cover too many areas of the problem (physiology, electrophysiological mechanisms, histology, therapies, medical imaging, mapping, biomarkers, models), and it seems that in some of them they do not go too deeply and do not have a large experience. There are sections that have very little impact on the overall structure of the article (physiopathological conditions, histology, blood biomarkers, anti-fibrotic drugs), which are likely to be of little appeal to the audience described above.

There are also sections that do not directly address the central topic of the review (fibrosis), such as 2.2 (Computed Tomography) and 2.4.1 (Voltage Mapping). In this latter, it would be appreciated if the authors could focus on the aspects that link voltage mapping with fibrotic infiltration, beyond a general review of these techniques. There are also sections that report information that is outside the scope of the section (CT: lines 271-275). And there are sections that are not well organized (5.2 is between two similar sections with different subject matter).

Regarding the content, I was surprised that a literature review on atrial fibrotic infiltration and its relationship with AF does not include any reference to the work by V. Fedorov et al. This group is the only one reporting in human hearts the relationship between fibrosis and AF mechanisms, using techniques considered as gold standard (optical mapping, high-resolution MRI, histology, etc.). The manuscript should address the relationship between interstitial and transmural fibrosis, fiber direction and the different mechanisms of AF maintenance (pubmed.ncbi.nlm.nih.gov/25984605/; pubmed.ncbi.nlm.nih.gov/26059724/; pubmed.ncbi.nlm.nih.gov/26671938/; pubmed.ncbi.nlm.nih.gov/29159313/; pubmed.ncbi.nlm.nih.gov/28862969/). I was also surprised that no reference was made to models of endo-epi dissociation, which have been the basis for different theories of AF maintenance (breakthroughs) based on fibrosis infiltration, as well as for the development of bilayer computational models used by different research groups (pubmed.ncbi.nlm.nih.gov/32634027/; pubmed.ncbi.nlm.nih.gov/25086270/; pubmed.ncbi.nlm.nih.gov/39576793/; pubmed.ncbi.nlm.nih.gov/32153419/; pubmed.ncbi.nlm.nih.gov/30485346/).

Regarding the use of other substrate features to guide AF ablation, such as wall thickness, the clinical trial conducted by Berruezo et al. should be mentioned (pubmed.ncbi.nlm.nih.gov/34480548/). And for mentioning that EAM can capture wall thickness, please include a specific reference, as (Abeln et al., 2021) refers to a general review. It looks also strange to discuss about DADs as potential atrial fibrillation mechanisms, when these have been reported in ventricular arrhythmias, as attested by the 3 references included in this section: (Xie et al., 2010) (Fujiwara etal., 2008) (Sato et al., 2009).

Finally, the section on modeling, surely where the authors have more experience, is quite biased towards the work of the research group formed by the authors of the paper. This section should have more variety in terms of references to works by other groups that do cardiac computational models with fibrotic infiltration (Blanca Rodriguez, Olaf Dossel & Axel Loewe, Natalia Trayanova & Patrick Boyle, etc.). For example, the text accurately reproduces the way of simulating fibrotic infiltration used by the authors (lines 606-634), but does not describe or compare with other methods in the literature. It would also appreciated if this section addressed important points of fibrotic modelling, such as the relationship between LGE-MRI intensity and the biophysical models used, such as in (pubmed.ncbi.nlm.nih.gov/35089057), or the use of other methods to simulate fibrosis, such as by coupling fibroblast cellular models (pubmed.ncbi.nlm.nih.gov/17959690/).

On the figures, looks very rare that Figure 5 includes a case/figure regarding atrioventricular dissociation and HFS, which is not addressed or described in the main text. In addition, resolution of Figure 3 should be improved for the final version.

REQUIRED ITEMS

- Please include an Abstract Figure file, as well as the Figure Legend text within the main article file. The Abstract Figure is a piece of artwork designed to give readers an immediate understanding of the Review Article and should summarise the main conclusions. If possible, the image should be easily 'readable' from left to right or top to bottom. It should show the physiological relevance of the Review so readers can assess the importance and content of the article. Abstract Figures should not merely recapitulate other figures in the Review. Please try to keep the diagram as simple as possible and without superfluous information that may distract from the main conclusion of the Review. Abstract Figures must be provided by authors no later than the revised manuscript stage and should be uploaded as a separate file during online submission labelled as File Type 'Abstract Figure'. Please ensure that you include the figure legend in the main article file. All Abstract Figures will be sent to a professional illustrator for redrawing and you may be asked to approve the redrawn figure before your paper is accepted.

- Your MS must include a complete "Additional information section" with the following 4 headings and content:

Competing Interests: A statement regarding competing interests. If there are no competing interests, a statement to this effect must be included. All authors should disclose any conflict of interest in accordance with journal policy.

Author contributions: Each author should take responsibility for a particular section of the study and have contributed to writing the paper. Acquisition of funding, administrative support or the collection of data alone does not justify authorship; these contributions to the study should be listed in the Acknowledgements. Additional information such as 'X and Y have contributed equally to this work' may be added as a footnote on the title page.

It must be stated that all authors approved the final version of the manuscript and that all persons designated as authors qualify for authorship, and all those who qualify for authorship are listed.

Funding: Authors must indicate all sources of funding, including grant numbers. If authors have not received funding, this must be stated.

It is the responsibility of authors funded by RCUK to adhere to their policy regarding funding sources and underlying research material. The policy requires funding information to be included within the acknowledgement section of a paper. Guidance on how to acknowledge funding information is provided by the Research Information Network. The policy also requires all research papers, if applicable, to include a statement on how any underlying research materials, such as data, samples or models, can be accessed. However, the policy does not require that the data must be made open. If there are considered to be good or compelling reasons to protect access to the data, for example commercial confidentiality or legitimate sensitivities around data derived from potentially identifiable human participants, these should be included in the

statement.

Acknowledgements: Acknowledgements should be the minimum consistent with courtesy. The wording of acknowledgements of scientific assistance or advice must have been seen and approved by the persons concerned. This section should not include details of funding.

- Please upload separate high quality figure files via the submission form.

- Author profile(s) must be uploaded via the submission form. Authors should submit a short biography (no more than 100 words for one author or 150 words in total for two authors) and a portrait photograph of the two leading authors on the paper. These should be uploaded and clearly labelled together in a Word document with the revised version of the manuscript. Any standard image format for the photograph is acceptable, but the resolution should be at least 300 DPI and preferably more. A group photograph of all authors is also acceptable, providing the biography for the whole group does not exceed 150 words.

END OF COMMENTS

FIBROSIS LITERATURE REVIEW COMMENTS

REFEREE COMMENTS

We sincerely thank the reviewers for their detailed and insightful feedback on our manuscript. Their suggestions have significantly improved the clarity and overall quality of our work. This document presents a point-by-point explanation for each modification. We have carefully updated the manuscript in accordance with the reviewer's recommendations.

Referee #1:

The authors provide a broad overview of the many aspects that play a role in the assessment and impact of atrial fibrosis, from mechanisms that lead to (different types of) fibrosis, techniques to quantify fibrosis (non)-invasively, the treatment options with respect to fibrosis (prevention, ablation,...), and computational tools that incorporate fibrosis to personalise treatment (digital twins) and to perform in-silico trials that consider structural remodelling.

Review

In general, the manuscript is well-written, and well-structured. The title (Mapping Techniques for Evaluating Fibrosis in Patients with Atrial Fibrillation) suggests that the review is primarily about techniques to assess the presence and degree of fibrosis in patients. The main text however also deals with several other aspects (mechanisms, treatment, computational modelling) of atrial fibrosis in detail. Perhaps a different title will better cover the content.

We appreciate the suggestion to alter the title to better reflect the content of the manuscript. We have changed the title to:

"Atrial fibrosis in Atrial Fibrillation: Mechanisms, Mapping Techniques, and Clinical Applications"

Alternatively, one could consider shortening the sections on mechanisms and therapy not related to mapping of fibrosis, and focus more on mapping and computational modelling.

We thank the reviewer for this feedback, we have shortened the sections to provide a better balance. Section 2 – Mapping Techniques for Detecting Fibrosis in AF was modified; the following subsections were modified/restructured: Sections 2.3, 2.4, 2.5, and Section 5. Also, Subsections 2.2 - Computed Tomography and 3.1 - Anti-fibrotic drugs were removed from the manuscript. We believe this has improved the readability and structure of the manuscript.

Specific remarks

1. Role of fibrosis

Arrhythmogenic Effects of Fibrotic Remodelling: consider to discuss the role of fibrosis in the development of endo-epicardial dissociation (see for instance doi: 10.1161/CIRCEP.113.000342)

We thank the reviewer for this request and have added the following paragraph in *Section 1.3 Arrhythmogenic Effects of Fibrotic Remodelling*:

"Atrial fibrosis plays a significant role in the development of endo-epicardial dissociation (EED) by disrupting the structural and electrical coupling between the endocardium and epicardium leading to an increase in fibrillation complexity. Fibrosis, particularly in the epicardial layer, disrupts electrical connection between muscle bundles, causing both intra-epicardial and EED. It also impairs transmural conduction, further developing EED. Additionally, endomysial fibrosis in the epicardial layer leads to loss of continuity, reducing the synchronizing effect, and increasing the complexity of fibrillatory conduction (Verheule *et al.*, 2014; Gharaviri *et al.*, 2020). A study by Eckstein et al., 2013 investigated the mechanisms underlying breakthrough events during AF, focusing on the role of transmural conduction and EED. The study demonstrated how fibrosis can disrupt electrical coupling between endocardial layers, enhancing EED, resulting in asynchronous activation and promoting AF persistence. Their study also demonstrated how the presence of fibrosis in the epicardial layer is associated with higher incidence of transmural conduction breakthrough, further contributing to the complex activation patterns observed during AF (Eckstein *et al.*, 2013)."

2. Mapping techniques

- Section 2.3.1: in lines 335 the authors paint a bleak picture of using LGE-CMR to quantify atrial fibrosis, by listing all the factors that may affect the quantification. Are there any efforts to standardize the methodology, apart from automated segmentation, and move the field forward?

We thank the reviewer for noticing this section is particularly negative – we have extended the section to be more balanced. Indeed, there are many factors that can affect the quantification of atrial fibrosis using LGE-CMR, and several ongoing efforts have been made to standardize the methodology apart from automated segmentation. Standardizing LGE-CMR can help personalize treatment strategies for patients with AF and allow the clinician to identify the best treatment approach. We have included in the manuscript different studies which proposed different standardizing approaches to quantify fibrosis in LGE-CMR, as well as a study on reproducibility and references to novel developments in imaging modalities. The paragraph can be found in Section 2.2.1 Quantifying Thresholds of Fibrosis and its Challenges:

"To mitigate these challenges, a study by Nairn et al. proposed the Estimated Optimized Image Intensity Threshold (EOITT) methods (Nairn *et al.*, 2023). This approach improved concordance between LGE-CMR and low-voltage substrate diagnosis from electroanatomical mapping, and enhanced prediction of rhythm outcomes after PVI. While the EOIIT method demonstrated improved sensitivity and specificity, there remained inconsistencies with EAM, especially on the posterior wall, emphasizing the need for further standardization to improve reproducibility and clinical application. Sim et al., demonstrated reproducibility of atrial fibrosis assessment using an open-source platform (Sim *et al.*, 2019). Novel developments in imaging modalities are discussed in the Future Directions Section of the review."

We have also added the following paragraph in Section 5.3 Improved Monitoring of Structural Remodelling:

"Assessing and tracking atrial fibrosis progression over time remains a significant challenge in AF management. Although LGE-CMR is an essential technique for visualizing atrial fibrosis, it has its limitations. To overcome these limitations, advanced imaging modalities need to be explored, which offer high-resolution images and a more detailed assessment of atrial structural remodeling. Higher resolution MRI techniques which allow higher spatial resolution and faster acquisition times, and detailed representation of atrial tissue, could potentially improve detection and quantification of fibrosis. Additionally, research into new contrast agents that enhance the contrast-to-noise ratio and specificity of atrial fibrosis can further improve the precision of LGE-CMR (Siebermair *et al.*, 2017; Cochet *et al.*, 2018; Luetkens *et al.*, 2018; Zghaib & Nazarian, 2018). Advanced non-invasive techniques, such as myocardial T1 mapping, show promise for evaluating myocardial fibrosis. This technique allows for a safer non-invasive assessment of the dynamics of fibrosis in myocardial atrial tissue, without the use of contrast agents, further enhancing atrial fibrosis detection, and management in patients with AF (Moon *et al.*, 2013; Taylor *et al.*, 2016)."

Section 2.4.1.3: Omnipolar mapping: are there any limitations for this technique that are worth mentioning, such as dependence on catheter configuration / proprietary algorithms?

We thank the reviewer for this question. The Reviewer makes an excellent point. Even though omnipolar mapping is a methodology that can be applied offline using EGMs from any available mapping system, currently the only mapping system that has this methodology incorporated into their system is EnsiteX (Abbott). Thereby, its use in generating voltage maps live during AF procedures is reliant on using proprietary technology. Further to this, the omnipolar mapping methodology available in Ensite X is dependent on the use of the HD grid catheter because of the configuration of the electrode positions and spacing. Thus, it is currently not feasible to apply the methodology to other catheters such as circular mapping catheters. We have updated *Section 2.3.1.3 Omnipolar Mapping* to include the limitations omnipolar mapping possesses, such as dependence on catheter configuration and proprietary algorithms.

“While omnipolar mapping offers many advantages, it also has its limitations. A study by Deno et al., highlighted a key assumption in omnipolar mapping, that the catheter is positioned on a flat myocardium. However, this assumption may not hold in curved or trabeculated areas, potentially affecting catheter orientation and accuracy (Deno *et al.*, 2017). Additionally, omnipolar mapping faces challenges related to algorithm implementation. Some systems restrict its use to specific proprietary catheters or software, and assumptions about wavefront propagation may not always be valid in complex scarred cardiac tissue (Karatela *et al.*, 2022; Dittrich *et al.*, 2024). While omnipolar mapping can be applied offline using EGMs from any mapping system, EnsiteX (Abbott) is currently the only system integrating it in real time. Moreover, its implementation in EnsiteX depends on the HD Grid catheter making it incompatible with other catheter types.”

- Section 2.5: here, the atrial-specific blood biomarker BMP10 is missing from the discussion (see for instance doi: 10.1016/j.jacep.2024.03.003)

We thank the reviewer for this request and have added the following paragraph in *Section 2.4 Blood Biomarkers of Atrial Fibrosis*:

“Recent research demonstrated that increased levels of serum bone morphogenic protein 10 (BMP10) were associated with persistent AF history (Winters *et al.*, 2024), as well as providing a marker for increased risk of ischemic stroke and mortality (Hijazi *et al.*, 2023; Hennings *et al.*, 2023). Winters et al., 2024 also showed that increased levels of BMP10 were correlated with higher incidences of late postoperative AF and left atrial

appendage endomysial fibrosis. However further research on the role of BMP10 in atrial fibrosis and its effect regulating cellular mechanisms is needed to further mechanistic understanding (Winters *et al.*, 2024)."

4. Digital twins

- This section would benefit from better structuring, better balance between general description and details.

We thank the reviewer for this feedback, we have modified section 4 – Digital Twins: Personalised Cardiac Modelling to provide a better balance, including more studies that model different components of fibrotic remodelling.

- Address limitations of digital twin modelling and how to mitigate/disseminate model uncertainty.

We thank the reviewer for this request, and have added the following paragraph in *Section 4: Digital Twins: Personalized Cardiac Modelling*:

"The use of digital twins in personalized cardiac modelling possesses many advantages as explained above. However, there are some limitations and areas of uncertainty when developing these models. Some personalised cardiac models focus on specific aspects of cardiac tissue physics which make it challenging to represent the complex arrhythmogenic substrates, such as modelling fibrosis or areas of necrotic infarcts (Connolly & Bishop, 2016; Niederer *et al.*, 2019; Sánchez & Loewe, 2022). Parameter sensitivity can lead to significant prediction difference, and developing accurate parametrization can be challenging (Deng *et al.*, 2017). Additionally, some models lack the spatial and temporal resolution needed to capture 3-dimensional dynamics (Lopez-Perez *et al.*, 2019). Validation remains a challenge due to limited experimental data available, and model credibility assessment methods are still evolving (Galappaththige *et al.*, 2022). Computational intensity is also a limitation, as high-resolution simulations require significant computational resources, but reduced-order or simplified eikonal models have been proposed to improve feasibility (Alberto Barrios Espinosa *et al.*, 2022). Uncertainty in EAM data collection and model equations can limit accuracy, especially when extrapolating different conditions (Wallman *et al.*, 2014; Clayton *et al.*, 2020). Furthermore, the current models do not account for interactions with other pathological conditions. Lack of knowledge of cardiac electrophysiology, including mechanisms like fibroblast coupling and mechano-transduction, further restrict model precision (Trayanova *et al.*, 2024). Patient-specific variability adds another layer of complexity, as general models, may not fully represent individual differences, encouraging the development of population-based virtual cohorts (Niederer *et al.*, 2020;

Roney *et al.*, 2022). Finally, ethical and practical challenges, including the need for accurate clinical data and regulatory approval, must be addressed before these models can be widely implemented."

5. Future directions

The discussion here is rather one-sided, focusing mostly on a selection of computational approaches/tools that are currently developed or tested. Other topics could be the development of new diagnostic markers, early detection, and improved monitoring of progression/regression of structural remodeling.

We thank the reviewer for this comment, and have added two extra sections in the Future Directions to address the suggested topics (Section 5.1 Advancements in Biomarkers and Early Detection, and Section 5.3 Improved Monitoring of Structural Remodelling). The following content has been added:

"Section 5.1 Advancements in Biomarkers and Early Detection

Early detection of atrial fibrosis is crucial for improving treatment outcomes in AF. Early detection of atrial fibrosis is crucial for improving treatment outcomes in patients with AF. Current diagnostics approaches rely strongly on imaging modalities such as LGE-CMR, which are effective but may not always be accessible or feasible for routine clinical use (Zhang *et al.*, 2024). As an alternative approach, there is current interest on biomarkers that are involved in extracellular matrix remodeling, inflammation, and fibrotic activity (Angeli *et al.*, 2024; Schotten *et al.*, 2024). Such biomarkers have been explored for their association with atrial fibrosis in patients with AF (see '*Section 2.4 Blood Biomarkers of Atrial Fibrosis*'). These biomarkers, when combined with clinical parameters, can provide a minimally invasive means of risk stratification and early detection of fibrotic remodeling (Ding *et al.*, 2020; Begg *et al.*, 2020; Koniari *et al.*, 2021). AI-driven models involving biomarker data with imaging modalities, could be used as potential tools to predict the risk of AF in patients.

Machine learning techniques have been used to develop multimodal predictive models incorporating electrocardiographic signatures of atrial fibrosis, such as p-wave indices and fragmentation, with clinical and imaging data to improve AF risk stratification (Filos *et al.*, 2017; Razeghi *et al.*, 2020; Luongo *et al.*, 2021; Sánchez *et al.*, 2021*a*). Future research should focus on validating these approaches on large-scale datasets, and assessing their utility in guiding personalized therapy. Additionally, further research is needed to evaluate the dynamic alterations in these biomarkers over time and their association with fibrosis progression, which could further enhance early detection and provide better treatment approaches for patients with AF.

5.3 Improved Monitoring of Structural Remodelling

Assessing and tracking atrial fibrosis progression over time remains a significant challenge in AF management. Although LGE-CMR is an essential technique for visualizing atrial fibrosis, it has its limitations. To overcome these limitations, advanced imaging modalities need to be explored, which offer high-resolution images and a more detailed assessment of atrial structural remodeling. Higher resolution MRI techniques which allow higher spatial resolution and faster acquisition times, and detailed representation of atrial tissue, could potentially improve detection and quantification of fibrosis. Additionally, research into new contrast agents that enhance the contrast-to-noise ratio and specificity of atrial fibrosis can further improve the precision of LGE-CMR (Siebermair *et al.*, 2017; Cochet *et al.*, 2018; Luetkens *et al.*, 2018; Zghaib & Nazarian, 2018). Advanced non-invasive techniques, such as myocardial T1 mapping, show promise for evaluating myocardial fibrosis. This technique allows for a safer non-invasive assessment of the dynamics of fibrosis in myocardial atrial tissue, without the use of contrast agents, further enhancing atrial fibrosis detection, and management in patients with AF (Moon *et al.*, 2013; Taylor *et al.*, 2016)."

Referee #2:

The authors present an interesting review focusing on an important topic in the field of atrial electrophysiology. They make a great review of atrial fribrosis, covering from the physiological point of view to its application in computational models. The article is very interesting for an audience focused on AF mapping and/or computational modelling, which is welcomed for those working in this field as it nicely links the patho-phisiology of fibrosis infiltration with mapping and computational models.

However, reading it gives the feeling that the authors have tried to cover too many areas of the problem (physiology, electrophysiological mechanisms, histology, therapies, medical imaging, mapping, biomarkers, models), and it seems that in some of them they do not go too deeply and do not have a large experience. There are sections that have very little impact on the overall structure of the article (physiopathological conditions, histology, blood biomarkers, anti-fibrotic drugs), which are likely to be of little appeal to the audience described above.

We thank the reviewer for this suggestion. We have reduced the text and combined these sections to instead direct the interested reader to other papers for more information. The sections that we modified are:

Sections 1.2, 1.3, 1.4,

Sections 2.1, 2.2 (removed), 2.3, 2.4, 2.5,

Sections 3.1 (removed), 3.2,

Sections 4

Sections 5.1, 5.3,

In Section 5 we included 2 new sections (Advancements in Biomarkers and Early Detection, Improved Monitoring of Structural Remodelling) and combined existing sections into a single section on modelling (In-Silico trial to Inform Ablation Therapy).

We believe this has improved the readability of the manuscript.

There are also sections that do not directly address the central topic of the review (fibrosis), such as 2.2 (Computed Tomography) and 2.4.1 (Voltage Mapping). In this latter, it would be appreciated if the authors could focus on the aspects that link voltage mapping with fibrotic infiltration, beyond a general review of these techniques. There are also sections that report information that is outside the scope of the section (CT: lines 271-275). And there are sections that are not well organized (5.2 is between two similar sections with different subject matter).

We thank the reviewer for this feedback, we have modified the section to provide a better balance. We have decided to remove Section 2.2 - Computed Tomography to reduce the length of the manuscript and improve its focus. We have removed any sections on voltage mapping that are not focused on fibrotic infiltration. Section 5.1 was removed, and Section 5.2 was moved and combined into Section 5.4 on in silico trials, so we believe the future work section is now better structured.

Regarding the content, I was surprised that a literature review on atrial fibrotic infiltration and its relationship with AF does not include any reference to the work by V. Fedorov et al. This group is the only one reporting in human hearts the relationship between fibrosis and AF mechanisms, using techniques considered as gold standard (optical mapping, high-resolution MRI, histology, etc.).

We thank the reviewer for this comment. The reviewer is correct with the following request, Fedorov et al’s work is indeed an important contribution to the understanding of

the relationship between atrial fibrosis and AF. We appreciate this comment, therefore have added the following in *Section 1.3 Arrhythmogenic Effects of Fibrotic Remodelling* including Fedorov et al's work in the manuscript to provide a more comprehensive perspective on this important topic.

"To gain a better understanding of the relationship between atrial fibrosis and AF, Fedorov et al provide significant insight using advanced techniques such as optical mapping, high-resolution MRI, and histology. One study highlighted the limitations of current AF treatments and the potential 3D computer models to optimize ablation strategies, demonstrating that human-derived models could improve the personalization and effectiveness of AF treatment approaches (Zhao *et al.*, 2015*b*). Their follow-up research further demonstrated how fibrosis affects electrophysiological properties, with high-resolution MRI revealing detailed structural changes (Zhao *et al.*, 2015*a*). Furthermore, Hansen et al revealed that intramural re-entry, anchored to fibrosis-insulated atrial bundles, drives sustained AF (Hansen *et al.*, 2017). This research highlights how 3D structural functional mapping exhibits significant differences in endocardial and epicardial activation, underlying the important role of intramural re-entry in AF. Additionally, further work of Fedorov et al., highlights the role of fibrotic tissue in altering conduction pathways, providing important information into the mechanism that drive fibrosis in AF in human hearts (Hansen *et al.*, 2017; Zhao *et al.*, 2017). Overall, this research emphasizes the significance of fibrotic infiltration in AF pathophysiology and the importance of advanced imaging techniques in studying these alterations."

The manuscript should address the relationship between interstitial and transmural fibrosis, fiber direction and the different mechanisms of AF maintenance (pubmed.ncbi.nlm.nih.gov/25984605/; pubmed.ncbi.nlm.nih.gov/26059724/; pubmed.ncbi.nlm.nih.gov/26671938/; pubmed.ncbi.nlm.nih.gov/29159313/; pubmed.ncbi.nlm.nih.gov/28862969/).

We appreciate the reviewer for this request. We recognize the importance of these connections and therefore have expanded in our manuscript to include these relationships Additionally, we have extended our manuscript to include fiber direction, and the different mechanisms in AF maintenance.

Addition of relationship between interstitial and transmural fibrosis in *Section 1.2 Different Types of Fibrosis*:
"Transmural fibrosis is a more advanced type of fibrosis, especially in the subepicardial and midwall layers. This type of fibrosis extends through the entire thickness of the atrial wall and can result in conduction disturbances and developing a higher risk of AF (Eckstein *et al.*, 2013; Ravelli *et al.*, 2023). This scarring across the atrial wall can result in significant conduction block, slowing electrical signals, which can cause the formation

of re-entry circuits sustaining AF. Additionally, transmural fibrosis causes electrical heterogeneity, which may cause AF to be more persistent."

Addition of fiber direction in atrial fibrosis in *Section 1.3 Arrhythmogenic Effects of Fibrotic Remodelling*:

"Furthermore, fibrosis develops along the anisotropic fiber orientation of the atria, creating conduction heterogeneity, and promoting re-entry circuits. Alteration of fiber orientations result in decreased anisotropy, indicating a disruption in the organized alignment of fibers in the atria, and slower conduction speeds due to these fibrotic regions (Kwan *et al.*, 2024). Additionally, in fibrotic tissue, increased fiber direction heterogeneity results in greater angle differences between neighboring fibers (Ho, 2002; Palacio *et al.*, 2021; Kwan *et al.*, 2024). In fibrotic areas, modified conduction directions may enable the development of unidirectional conduction block (Kwan *et al.*, 2024)."

Addition of different mechanisms of AF maintenance in *Section 1.3 Arrhythmogenic Effects of Fibrotic Remodelling*:

"AF maintenance is influenced by several mechanisms involving atrial fibrosis. It disrupts normal electrical conduction by forming fibrotic tissue that slows impulse propagation and re-entry circuits (Nguyen *et al.*, 2014; Verheule & Schotten, 2021). Fibrosis alters the expression of ion channels and affects the myocyte action potential, increasing the susceptibility to ectopic foci especially in the pulmonary veins, causing AF maintenance (Schotten *et al.*, 2024; Karakasis *et al.*, 2024). Inflammation also plays a role in promoting fibroblast activation collagen deposition (Nattel, 2017). Furthermore, atrial fibrosis can lead to atrial dilation, increasing the complexity of re-entry circuits due to the increase in the atrial surface area (de Groot *et al.*, 2024). These different mechanisms that affect atrial fibrosis contribute to AF maintenance."

I was also surprised that no reference was made to models of endo-epi dissociation, which have been the basis for different theories of AF maintenance (breakthroughs) based on fibrosis infiltration, as well as for the development of bilayer computational models used by different research groups (pubmed.ncbi.nlm.nih.gov/32634027/; pubmed.ncbi.nlm.nih.gov/25086270/; pubmed.ncbi.nlm.nih.gov/39576793/; pubmed.ncbi.nlm.nih.gov/32153419/; pubmed.ncbi.nlm.nih.gov/30485346/).

We thank the reviewer for this request. We have added a paragraph to our manuscript incorporating insights of the role of fibrosis in endo-epicardial dissociation found below from the previous comment from reviewer 1 also on endo-epicardial dissociation.

“Atrial fibrosis plays a significant role in the development of endo-epicardial dissociation (EED) by disrupting the structural and electrical coupling between the endocardium and epicardium leading to an increase in fibrillation complexity. Fibrosis, particularly in the epicardial layer, disrupts electrical connection between muscle bundles, causing both intra-epicardial and EED. It also impairs transmural conduction, further developing EED. Additionally, endomysial fibrosis in the epicardial layer leads to loss of continuity, reducing the synchronizing effect, and increasing the complexity of fibrillatory conduction (Verheule *et al.*, 2014; Gharaviri *et al.*, 2020). A study by Eckstein et al., 2013 investigated the mechanisms underlying breakthrough events during AF, focusing on the role of transmural conduction and EED. The study demonstrated how fibrosis can disrupt electrical coupling between endocardial layers, enhancing EED, resulting in asynchronous activation and promoting AF persistence. Their study also demonstrated how the presence of fibrosis in the epicardial layer is associated with higher incidence of transmural conduction breakthrough, further contributing to the complex activation patterns observed during AF (Eckstein *et al.*, 2013).”

But in addition to this specific comment with examples of papers, we have added the following paragraph into our manuscript addressing the use of different bilayer computational models used by different research groups in *Section 1.3 Arrhythmogenic Effects of Fibrotic Remodelling*:

“Experimental models of EED have been fundamental in informing different theories of AF maintenance, especially in relation to breakthroughs driven by fibrosis infiltration. These animal models provide critical insights into the complex mechanisms underlying AF and have motivated the development of bilayer computational models that incorporate interactions between the endocardium and epicardium. A study by Parameswaran et al., demonstrated significant heterogeneity in dissociation during fibrillation in patients with persistent AF, highlighting the need for simultaneous endo-epicardial mapping (Parameswaran *et al.*, 2020). Additionally, Verheule et al., 2014, demonstrated that epicardial fibrosis disrupts electrical connections, promoting dissociation and increasing AF stability (Verheule *et al.*, 2014). Computational models, such as Zafarghandi and Jacquemet 2024, revealed that fibrosis density and weaker interlayer coupling promote breakthrough waves, enhancing AF maintenance (Zakeri Zafarghandi & Jacquemet, 2024). Similarly, Gharaviri et al. 2020, found that epicardial fibrosis increases dissociation and breakthrough waves (Gharaviri *et al.*, 2020). Finally, Chen et al. 2018 revealed that intramural fibrosis and dissociation sustain AF, highlighting the importance of structural remodeling (Chen *et al.*, 2018).”

Regarding the use of other substrate features to guide AF ablation, such as wall

thickness, the clinical trial conducted by Berruezo et al. should be mentioned (pubmed.ncbi.nlm.nih.gov/34480548/).

We thank the reviewer for highlighting this interesting clinical trial. We have removed the discussion of using CT features for guiding AF ablation to improve the focus of the review so unfortunately have not included this trial.

And for mentioning that EAM can capture wall thickness, please include a specific reference, as (Abeln et al., 2021) refers to a general review.

We thank the reviewer for the comment, and have added the following reference which mentions EAM modalities for assessing wall thickness:

https://pubmed.ncbi.nlm.nih.gov/35557022/

It looks also strange to discuss about DADs as potential atrial fibrillation mechanisms, when these have been reported in ventricular arrhythmias, as attested by the 3 references included in this section: (Xie et al., 2010) (Fujiwara etal., 2008) (Sato et al., 2009).

We appreciate the reviewer for this comment. To avoid any confusion and to focus on atrial rather than ventricular arrhythmias, we have added references specific to the atria when discussing DAD as shown in the paragraph below (found in *Section 1.3 Arrhythmogenic Effects of Fibrotic Remodeling):*

"In addition, fibrotic remodelling triggers ectopic activity by disrupting the protective source–sink mismatch that normally prevents small groups of misbehaving myocytes from triggering propagating impulses (Sánchez *et al.*, 2021*b*). In healthy myocardium, spontaneous calcium waves or delayed afterdepolarizations (DADs) in isolated myocytes fail to generate detectable voltage changes unless a critical mass of myocytes depolarizes synchronously (Voigt *et al.*, 2012)."

Finally, the section on modeling, surely where the authors have more experience, is quite biased towards the work of the research group formed by the authors of the paper. This section should have more variety in terms of references to works by other groups that do cardiac computational models with fibrotic infiltration (Blanca Rodriguez, Olaf Dossel & Axel Loewe, Natalia Trayanova & Patrick Boyle, etc.). For example, the text accurately reproduces the way of simulating fibrotic infiltration used by the authors (lines 606-634), but does not describe or compare with other methods in the literature. It would also appreciated if this section addressed important points of fibrotic modelling, such as the relationship between LGE-MRI intensity and the biophysical models used, such as in (pubmed.ncbi.nlm.nih.gov/35089057), or the use of other methods to

simulate fibrosis, such as by coupling fibroblast cellular models (pubmed.ncbi.nlm.nih.gov/17959690/).

We thank the reviewer for the request. We appreciate your point about including more diverse references in the modeling section. We have added the paragraph below, which incorporates additional references with methods from other researchers, such as Olaf Dossel, Axel Loewe, Natalia Trayanova and Patrick Boyle, to provide a more balanced view. We agree that addressing the relationship between LGE-CMR intensity and biophysical models, as well as incorporating other methods to simulate fibrosis, such as coupling fibroblast cellular models, would significantly enhance the manuscript. Thank you for the reference provided, we have also added them to the paragraph below (found in Section 4)

“Several studies have incorporated the effects of replacement fibrosis or interstitial fibrosis on wavefront propagation and electrogram. In volumetric models, fibrosis is often increased on the epicardium, and atrial fibrosis is more pronounced in the subepicardial layers than in the endocardial muscle bundles (Vigmond *et al.*, 2016; Masè *et al.*, 2024). A study by Balaban et al. 2020, developed a computational method to simulate life-threatening arrhythmias in non-ischemic cardiomyopathy by modelling interstitial fibrosis and replacement fibrosis to investigate how the modelling methodology for fibrosis influences arrhythmia mechanisms (Balaban *et al.*, 2020; Myklebust *et al.*, 2024). Furthermore, the effect of fibroblast coupling to myocytes can be modelled using several fibroblast cell models with different fibroblast densities resulting in different electronic effects (Jacquemet & Henriquez, 2007; Sánchez *et al.*, 2019; Colman *et al.*, 2023). Fibrosis can also be modelled at the cellular level by modifying ionic conductances to represent its effect on electrophysiology (Zahid *et al.*, 2016). Additionally, the models incorporating ionic changes in fibrotic remodeling can also be combined with conductivity changes. For example, in the modelling methodology of the OPTIMA trial conductivity values are reduced with alterations in the anisotropy ratio, representing interstitial fibrosis, gap junction remodeling and higher disruption of cell-to-cell coupling in the transverse direction of cardiac fibers, together with modifications at the cellular level. A review by Sánchez and Loewe 2022 emphasized how multiscale models facilitate the analysis of the different mechanisms driving complex fractionated atrial electrograms and the detection of fibrosis in electrograms (Sánchez & Loewe, 2022).”

We have also added use of calibration models in *Section 5.4 In-Silico Trials to Inform Ablation Therapy*:
“Future directions for patient-specific biophysical modelling include refining uncertainty quantification methodologies for rapid calibration tor patient-specific data (Martínez Díaz *et al.*, 2024)”

On the figures, looks very rare that Figure 5 includes a case/figure regarding atrioventricular dissociation and HFS, which is not addressed or described in the main text. In addition, resolution of Figure 3 should be improved for the final version.
We thank the reviewer for this request. A higher-resolution of Figure 3 has been updated and included into the manuscript. Figure 5 was changed to a different figure which shows a better representation of an EAM system, specifically EnsiteX (Abbott) mapping system.

REQUIRED ITEMS

- Please include an Abstract Figure file, as well as the Figure Legend text within the main article file. The Abstract Figure is a piece of artwork designed to give readers an immediate understanding of the Review Article and should summarise the main conclusions. If possible, the image should be easily 'readable' from left to right or top to bottom. It should show the physiological relevance of the Review so readers can assess the importance and content of the article. Abstract Figures should not merely recapitulate other figures in the Review. Please try to keep the diagram as simple as possible and without superfluous information that may distract from the main conclusion of the Review. Abstract Figures must be provided by authors no later than the revised manuscript stage and should be uploaded as a separate file during online submission labelled as File Type 'Abstract Figure'. Please ensure that you include the figure legend in the main article file. All Abstract Figures will be sent to a professional illustrator for redrawing and you may be asked to approve the redrawn figure before your paper is accepted.

New Abstract Figure guides the reader through the paper structure.

- Your MS must include a complete "Additional information section" with the following 4 headings and content:

Competing Interests: A statement regarding competing interests. If there are no competing interests, a statement to this effect must be included. All authors should disclose any conflict of interest in accordance with journal policy.

No competing interests for Laura Bevis

No competing interests for Caterina Vidal Horrach

No competing interests for Cynthia Nwanna

No competing interests for Semhar Biniam Misghina

No competing interests for Mahmoud Ehnesh

No competing interests for Alexander Zolotarev

No competing interests for Sayed Al-Aidorous

No competing interests for Shohreh Honarbakhsh

No competing interests for Caroline Roney

Author contributions: Each author should take responsibility for a particular section of the study and have contributed to writing the paper. Acquisition of funding, administrative support or the collection of data alone does not justify authorship; these contributions to the study should be listed in the Acknowledgements. Additional information such as 'X and Y have contributed equally to this work' may be added as a footnote on the title page.

CVH & CHR conceptualized the review. CVH wrote and edited the manuscript, and generated figures for all sections of the review. LB contributed to the writing (sections: 1.2, 2.2, 2.3, 2.4, 3.2, 4, 5.1, 5.3), editing, and generation of figures in biorender in all sections. LB contributed to figure 1, and 9; CVH contributed to figure 2, 9, and Abstract Figure. CHR contributed extensively to the writing and editing process, refining the manuscript's clarity and consistency while ensuring scientific accuracy. Additionally, CHR helped structure arguments, enhancing the logical flow of the discussions, and aligning the manuscript with recent research. CN contributed to the writing of section 1.1 Pathophysiology of atrial fibrosis and 1.3 Arrhythmogenic effects of fibrotic remodeling, and generation of figure 3. ME contributed to the writing of section 2.3.1 (quantifying thresholds of fibrosis and its challenges). SBM contributed to the generation of figure 4. AZ contributed to the writing of section 5.4 In-silico trials to inform ablation therapy. SAA, SH, and CHR contributed to the design of the review and data presentation. All listed authors contributed to the work and read and approved the final manuscript.

It must be stated that all authors approved the final version of the manuscript and that all persons designated as authors qualify for authorship, and all those who qualify for authorship are listed.

Funding: Authors must indicate all sources of funding, including grant numbers. If authors have not received funding, this must be stated.

Dr. Caroline Roney funding - This research was funded by a UKRI Future Leaders Fellowship (MR/W004720/1).

Dr. Laura Bevis funding - The authors (LB specifically) acknowledge funding from Queen Mary University of London in contribution towards the Digital Europe Programme of the European Commission (grant agreement 101083771) for the EDITH project. We also acknowledge funding from the National Institute for Health Research Barts Biomedical Research Centre (NIHR203330).

Dr Shohreh Honarbakhsh funding - This study was funded by the British Heart Foundation Clinical Intermediate Fellowship FS/ICRF/22/26034

It is the responsibility of authors funded by RCUK to adhere to their policy regarding funding sources and underlying research material. The policy requires funding information to be included within the acknowledgement section of a paper. Guidance on how to acknowledge funding information is provided by the Research Information Network. The policy also requires all research papers, if applicable, to include a statement on how any underlying research materials, such as data, samples or models, can be accessed. However, the policy does not require that the data must be made open. If there are considered to be good or compelling reasons to protect access to the data, for example commercial confidentiality or legitimate sensitivities around data derived from potentially identifiable human participants, these should be included in the statement.

Acknowledgements: Acknowledgements should be the minimum consistent with courtesy. The wording of acknowledgements of scientific assistance or advice must have been seen and approved by the persons concerned. This section should not include details of funding.

We would like to thank all authors for their contributions to this review. Also, would like to express our gratitude to Digital Environment Research Institute (DERI), for a collaborative environment offered by the institute in the development of this work.

- Please upload separate high quality figure files via the submission form.

- Author profile(s) must be uploaded via the submission form. Authors should submit a short biography (no more than 100 words for one author or 150 words in total for two authors) and a portrait photograph of the two leading authors on the paper. These should be uploaded and clearly labelled together in a Word document with the revised version of the manuscript. Any standard image format for the photograph is acceptable, but the resolution should be at least 300 DPI and preferably more. A group photograph of all authors is also acceptable, providing the biography for the whole group does not exceed 150 words.

Caterina Vidal Horrach is a PhD student at Queen Mary University of London, focusing on mechanism-specific treatment approaches for atrial fibrillation using patient-specific modeling and artificial intelligence. She holds an MSc in Bioinformatics (Distinction) from Queen Mary University and a BSc in Biomedical Sciences from Oxford Brookes University. Caterina has contributed to several peer-reviewed publications in cardiac modeling and computational cardiology. She is also a PhD demonstrator, supporting undergraduate and MSc students in biomedical imaging and AI applications. Her research interests include computational modeling, cardiac electrophysiology, and AI-driven healthcare solutions.

Dear Dr Roney,

Re: JP-TR-2025-288680R1 "**Atrial Fibrosis in Atrial Fibrillation: Mechanisms, Mapping Techniques, and Clinical Applications**" by Caterina Vidal Horrach, Laura Bevis, Cynthia Nwanna, Alexander Zolotarev, Mahmoud Ehnesh, Semhar Biniam Misghina, Sayed Al-Aidarous, Shohreh Honarbakhsh, and Caroline H Roney

We are pleased to tell you that your paper has been accepted for publication in The Journal of Physiology.

IMPORTANT
We only seem to have separate figure files for three of the figures (figs 1, 3 and 9). Our typesetters will need a full set of figure files.
Please can you email the figures files to us as soon as possible at: jp@physoc.org

TRANSPARENT PEER REVIEW POLICY: To improve the transparency of its peer review process, The Journal of Physiology publishes online as supporting information the peer review history of all articles accepted for publication. Readers will have access to decision letters, including Editors' comments and referee reports, for each version of the manuscript, as well as any author responses to peer review comments. Referees can decide whether or not they wish to be named on the peer review history document.

The last Word (or similar) version of the manuscript provided will be used by the Production Editor to prepare your proof. When this is ready you will receive an email containing a link to Wiley's Online Proofing System. The proof should be thoroughly checked and corrected as promptly as possible.

Authors should note that it is too late at this point to offer corrections prior to proofing. Major corrections at proof stage, such as changes to figures, will be referred to the Editors for approval before they can be incorporated. Only minor changes, such as to style and consistency, should be made at proof stage. Changes that need to be made after proof stage will usually require a formal correction notice.

All queries at proof stage should be sent to: TJP@wiley.com.

Are you on Twitter? Once your paper is online, why not share your achievement with your followers? Please tag The Journal (@jphysiol) in any tweets and we will share your accepted paper with our 30,000 followers!

Yours sincerely,

Bjorn Knollmann
Senior Editor
The Journal of Physiology

P.S. - You can help your research get the attention it deserves! Check out Wiley's free Promotion Guide for best-practice recommendations for promoting your work at www.wileyauthors.com/eeo/guide. You can learn more about Wiley Editing Services which offers professional video, design, and writing services to create shareable video abstracts, infographics, conference posters, lay summaries, and research news stories for your research at www.wileyauthors.com/eeo/promotion.

IMPORTANT NOTICE ABOUT OPEN ACCESS: To assist authors whose funding agencies mandate public access to published research findings sooner than 12 months after publication, The Journal of Physiology allows authors to pay an Open Access (OA) fee to have their papers made freely available immediately on publication.

The Corresponding Author will receive an email from Wiley with details on how to register or log-in to Wiley Authors Services where you will be able to place an order.

You can check if your funder or institution has a Wiley Open Access Account here: https://authorservices.wiley.com/author-resources/Journal-Authors/licensing-and-open-access/open-access/author-compliance-tool.html.

EDITOR COMMENTS

Reviewing Editor:

All points were addressed satisfactorily.

Senior Editor:

The manuscript is now acceptable. Thank you for your excellent contribution to the Journal!

REFEREE COMMENTS

Referee #1:

I am pleased with the changes the authors made, which significantly improved the structure, focus, and readability of the manuscript. I have no further comments to make.

Referee #2:

The authors have responded very satisfactorily to all the questions raised, and the manuscript now presents a more scientifically balanced and potentially more attractive content. My congratulations to the authors, no further changes are proposed.
